# A novel signature based on CeRNA and immune status predicts prognostic risk and drug sensitivity in gastric cancer patients

**DOI:** 10.3389/fimmu.2022.951135

**Published:** 2022-11-22

**Authors:** Wei Cao, Weiguo Zhou, Mengying Li, Zehua Zhang, Xun Zhang, Kang Yang, Shiyi Yang, Guodong Cao, Bo Chen, Maoming Xiong

**Affiliations:** ^1^ Department of General Surgery, First Affiliated Hospital of Anhui Medical University, Hefei, China; ^2^ Laboratory for Reproductive Immunology, Hospital of Obstetrics and Gynecology, Shanghai Medical School, Fudan University, Shanghai, China; ^3^ Department of General Surgery, Anhui Public Health Clinical Center, Hefei, China; ^4^ Department of Surgery, The People’s Hospital of Hanshan County, Ma’anshan, China

**Keywords:** gastric cancer, CircRNA, immunocyte infiltration, competing endogenous RNA network, prognostic model, plasma variant translocation 1, mast cell

## Abstract

**Background:**

At present, there is increasing evidence that both competitive endogenous RNAs (ceRNAs) and immune status in the tumor microenvironment (TME) can affect the progression of gastric cancer (GC), and are closely related to the prognosis of patients. However, few studies have linked the two to jointly determine the prognosis of patients with GC. This study aimed to develop a combined prognostic model based on ceRNAs and immune biomarkers.

**Methods:**

First, the gene expression profiles and clinical information were downloaded from TCGA and GEO databases. Then two ceRNA networks were constructed on the basis of circRNA. Afterwards, the key genes were screened by univariate Cox regression analysis and Lasso regression analysis, and the ceRNA-related prognostic model was constructed by multivariate Cox regression analysis. Next, CIBERSORT and ESTIMATE algorithms were utilized to obtain the immune cell infiltration abundance and stromal/immune score in TME. Furthermore, the correlation between ceRNAs and immunity was found out through co-expression analysis, and another immune-related prognosis model was established. Finally, combining these two models, a comprehensive prognostic model was built and visualized with a nomogram.

**Results:**

The (circRNA, lncRNA)-miRNA-mRNA regulatory network of GC was constructed. The predictive power of ceRNA-related and immune-related prognosis models was moderate. Co-expression analysis showed that the ceRNA network was correlated with immunity. The integrated model of combined ceRNAs and immunity in the TCGA training set, the AUC values of 1, 3, and 5-year survival rates were 0.78, 0.76, and 0.78, respectively; in the independent external validation set GSE62254, they were 0.81, 0.79, and 0.78 respectively; in GSE15459, they were 0.84, 0.88 and 0.89 respectively. Besides, the prognostic score of the comprehensive model can predict chemotherapeutic drug resistance. Moreover, we found that plasma variant translocation 1 (PVT1) and infiltrating immune cells (mast cells) are worthy of further investigation as independent prognostic factors.

**Conclusions:**

Two ceRNA regulatory networks were constructed based on circRNA. At the same time, a comprehensive prognosis model was established, which has a high clinical significance for prognosis prediction and chemotherapy drug selection of GC patients.

## Introduction

More than 1 million patients worldwide are diagnosed with GC every year, of which more than 700,000 patients die (1). Currently, GC is the fourth most common cancer and the second leading cause of cancer death in the world, with more than 70% of cases occurring in developing countries (2, 3). In Asia, GC is the third most prevalent cancer (after breast cancer and lung cancer) and the second most common cause of death (after lung cancer) (4). Although the incidence and mortality rate of GC in China have been gradually decreasing in recent years, it is still a major public health problem (5). Since GC has no obvious symptoms or only some nonspecific symptoms in the early stage, the disease is easily ignored and misdiagnosed (6). Most patients are already at an advanced stage when diagnosed, and lose the best opportunity for surgical treatment. In addition, the drug resistance of chemotherapeutic drugs is serious, resulting in a poor prognosis for many patients (7, 8). Therefore, it is particularly important to explore the molecular mechanism of GC occurrence and development, find new prognostic markers, and further construct a prognostic model with superior accuracy and stable effect.

Circular RNAs (circRNAs) shape a covalently closed continuous loop which have no 5’-3’ polarity and contain no polyA tail (9, 10). Long noncoding RNAs (lncRNAs) are a class of untranslated RNA molecules, which are typically greater than 200 nucleotides in length and do not code for proteins (11). MicroRNAs (miRNAs) are non-coding single stranded RNA molecules with about 22 nucleotides encoded by endogenous genes, which can down-regulate the expression of related genes (12). In recent years, a large number of studies have found that many genes and non-coding RNAs (including circRNAs, lncRNAs, miRNAs) play a paramount role in the GC (13–18). At the same time, based on the hypothesis of competitive endogenous RNA (ceRNA) regulatory network, circRNAs and lncRNAs mainly regulate gene expression by adsorbing miRNAs or acting as miRNA response elements (MRE) (19, 20). It is restricted to understand the molecular mechanism of GC by a single gene or a single non-coding RNA. However, it may get a better choice to further explore the molecular mechanism of GC by combining a large number of genes and related non-coding RNAs through the ceRNA network. At the same time, based on the genes and non-coding RNAs in ceRNA, we can construct the prognosis model of GC and find out the independent prognostic markers to further supplement the prognosis prediction system of GC (15, 21). Therefore, the construction of ceRNA network and prognosis model is of great significance to the molecular mechanism and prognosis prediction of GC.

As a solid tumor, GC contains not only tumor cells but also infiltrating immune cells, stromal cells, epithelial cells, vascular cells, etc. (22). In the TME of GC, infiltrating immune cells (mainly including tumor infiltrating lymphocytes, tumor associated macrophages, dendritic cells, and bone marrow derived inhibitory cells) and stromal cells are two very important non-tumor cells. Numerous studies have shown that they are valuable for the occurrence and development, prognosis evaluation, and drug resistance evaluation of GC (23–26). However, it is quite difficult to directly measure the proportion of various cells in the tumor microenvironment to determine the purity and the abundance of infiltrating immune cells (27). We can solve the above problems through two algorithms: “cell type identification by estimating relative subsets of RNA transcripts” (CIBERSORT) and “Estimation of Stromal and Immune cells in MAlignant Tumour tissues using Expression data” (ESTIMATE). The CIBERSORT algorithm contains 547 genes, and uses the principle of linear support vector regression to deconvolute the expression matrix of immune cell subtypes to estimate the abundance of 22 immune cells (28). According to the specific gene expression characteristics of stroma and immune cells, the ESTIMATE algorithm can obtain the stromal/immune score through the tumor tissue transcription map to predict the tumor purity (29).

However, there are currently few studies linking ceRNA to the immune status in the tumor microenvironment, and further developing a comprehensive prognosis model for the prognosis evaluation and prediction of chemotherapy drug resistance in patients with GC. In this study, we constructed two ceRNA regulatory networks, circRNA-miRNA-mRNA and (circRNA, lncRNA)-miRNA based on circRNA, to further explore the molecular mechanism of GC. The abundance of infiltrating immune cells and stromal/immune score of GC microenvironment was obtained by ESTIMATE and CIBERSORT algorithm. At the same time, we constructed ceRNA-related and immune-related prognosis models respectively, and further combined them to construct a comprehensive prognosis model, which proposed a new method for prognosis assessment of GC patients. In addition, our comprehensive model can also predict the drug resistance of chemotherapeutic drugs, which provides a new idea for the selection of chemotherapy regimen for GC.

## Materials and methods

### Data collection

The RNAs expression data were derived from the Gene Expression Omnibus (GEO) database (http://www.ncbi.nlm.nih.gov/gds). GSE83521 analyzed circRNA through microarray, including six gastric cancer tissues and six matched adjacent tissues. The gastric cancer tissues were from patients with stage III gastric cancer. GSE78092 used non coding RNA microarray to analyze the differential expression of circRNAs in three pairs of gastric cancer and adjacent tissues, and the results were consistent with those verified by qPCR. GSE23739 analyzed the expression of miRNAs in 40 pairs of gastric cancer and normal gastric mucosa tissues from Singapore through Agilent Human miRNA Microarrays (V2). GSE29998 detected the RNA expression in 50 gastric cancer tissues and 49 normal gastric mucosa tissues through aflymetrix SNP arrays and Illumina mRNA expression arrays. The samples are from Russia and Vietnam. At the same time, we download the expression data and clinical data of GC from the Cancer Genome Atlas (TCGA) database (https://cancergenome.nih.gov). The expression data of mRNA and lncRNA included 407 samples (375 GC tissues, 32 normal tissues); miRNA expression data samples included 477 samples (436 GC tissues, 41 normal gastric tissues); and 443 samples Clinical data of GC (survival time, survival status, age, gender, T stage, N stage, M stage, clinical stage, pathological stage). In addition, the GC expression data and survival data in data sets GSE62254 and GSE15459 were collected from the GEO database as independent external validation sets. GSE62254 obtained the expression level of mRNAs in 300 gastric cancer patients in Asian Cancer Research Group (ACRG) through microarray analysis, and also collected the survival time, survival status, age, TNM stage and other clinical data of patients. GSE15459 obtained the mRNA expression level of 192 patients with primary gastric cancer (original 200 cases, excluding 8 cases in the original text) through microarray analysis. At the same time, the survival time, survival status, age, sex, TNM stage and other clinical information of patients were collected.

### Differential expression analysis of circRNAs, lncRNAs, miRNAs, and mRNAs

The limma package of R4.0.5 software was used to perform differential expression analysis on the RNA expression profiles downloaded from the GEO database and the TCGA database. The threshold was *P* < 0.05 and |log_2_FC| > 1. The differentially expressed RNAs shared by the two databases were used for the next analysis.

### Construction of circRNA-miRNA-mRNA and (circRNA, lncRNA)-miRNA ceRNA regulatory network

The intersection of differentially expressed circRNAs in GSE83521 and GSE78092 was taken for the following analysis. The miRNA targeted by circRNA was predicted by circbank database (http://www.circbank.cn/index.html). LncBasev.2 database (http://carolina.imis.athena-innovation.gr) was used to predict miRNA targeting lncRNA. Target Scan Human 7.2 Database (http://www.targetscan.org/vert_72), miRWalk database (http://mirwalk.umm.uni-heidelberg.de), and miRDB database (http://mirdb.org), and the three databases intersection was used to forecast the target mRNA of miRNA. Cytoscape 3.7.2 was used to construct circRNA-miRNA-mRNA and (circRNA, lncRNA)-miRNA regulatory networks. Then combine the above two ceRNA regulatory networks to construct a (circRNA, lncRNA)-miRNA-mRNA regulatory network.

### Gene enrichment analysis and protein interaction analysis in ceRNA network

Gene ontology (GO) annotation enrichment and Kyoto Encyclopedia of Genes and Genomes (KEGG) pathway analysis were carried out through the DAVID6.8 database (https://david.ncifcrf.gov), the threshold was *P* < 0.05. Protein-protein interaction (PPI) analysis was performed using STRING11.0 (https://string-db.org), requiring a composite score of > 0.4. The data was further imported into Cytoscape 3.7.2 according to the Degree algorithm to obtain the key genes with the highest correlation in the PPI network.

### Establishment of a prognostic model based on the ceRNA network

The expression data of mRNAs and lncRNAs were matched with clinical data, and the samples with missing data were deleted. Univariate Cox regression analysis was performed on mRNAs, lncRNAs, and clinical features by the survival package of R4.0.5 (threshold value *P* < 0.05). Furthermore, the glmnet package and survival package were used for Lasso regression analysis and multivariate Cox regression analysis to establish the prognosis model. Risk score= β*expRNA1 + β*expRNA2 +…+ β*age + β*gender +…+β*stage (Among them, β is the regression coefficient of the corresponding factor obtained by multivariate Cox regression analysis. ExpRNA is the expression of the corresponding RNA, and the clinicopathological features are converted into the corresponding numbers). Finally, the patients were divided into high-risk group and low-risk group according to the median risk scores. Then, Kaplan Meier (K-M) survival analysis was carried out in the high-risk group and low-risk group using the survival package (threshold *P* < 0.05). At the same time, the ROC curves of 3-year and 5-year overall survival rates were plotted by the survival and timeROC packages, and the area under the curve (AUC) of both were calculated. Finally, K-M survival analysis was performed on the genes in the model to find out the independent prognostic markers of GC.

### Obtaining the tumor microenvironment immunity through CIBERSORT and ESTIMATE algorithms

The gene expression profiles of GC downloaded from TCGA database and external validation sets GSE62254 and GSE15459 were input into the CIBERSORT algorithm to predict the abundance of 22 infiltrating immune cells in the tumor microenvironment, with a threshold of *P* < 0.05. At the same time, the ESTIMATE algorithm was used to obtain the stromal/immune scores of the above three data sets.

### Correlation analysis between ceRNA related prognostic models and immune status

First, GSEA was used to analyze the high and low-risk groups divided by ceRNA- related prognosis model, and the factors affecting the prognosis were obtained. The background file was c2.cp.kegg.v7.4.symbols.gmt, and the thresholds for all enrichment results were Nominal *p*-value < 0.05 and FDR *q*-value < 0.25. Then, ggplot2, ggpubr, ggExtra, and corrplot software packages were used to analyze the correlation of 22 infiltrating immune cells and stromal/immune scores with ceRNAs, with a threshold *P* < 0.05.

Meanwhile, the genes related to RCAN2 expression were found (correlation coefficient>0.5, P<0.05). The KEGG and GO enrichment analysis of the above genes was carried out to find out the potential mechanism of RCAN2 in gastric cancer. The next step is to further verify the signal pathway regulated by RCAN2 through correlation analysis. In addition, immunohistochemistry in HPA database was used to verify the expression level of related proteins at the protein level.

Then, similar methods were used to analyze the correlation between the risk score of the ceRNA-related prognosis model and immunity. Finally, using cBioPortal (http://www.Cbioportal.ORG) online database to analyze the mutation frequency and mutation type of all RNAs in the ceRNA-related model. And further found out the differences of the mutation types and frequencies of RNAs in different types of GC.

### Establishment of an immune related prognosis model

Similar to the above method of establishing the prognosis model, the prognosis model of GC was constructed based on the abundance of 22 infiltrating immune cells and the immune/stromal scores of the tumor microenvironment.

### Construction of a comprehensive prognostic model combining ceRNAs and immunity and verifying it with an external data set

Similar to the above, all the factors with *P* value less than 0.05 after multivariate Cox analysis in the above two models are integrated into the comprehensive model. Furthermore, the same integrated model was used for verification in the external validation sets GSE62254 and GSE15459. Finally, the final nomogram was drawn on the basis of the comprehensive model. In addition, we conducted subgroup analysis on TCGA gastric cancer patients and divided them into Microsatellite Facility (MSI) group and Microsatellite Stability (MSS) group. MSI group can also be divided into MSI-H and MSI-L groups. Further study the correlation between risk grouping and microsatellite. Finally, we also analyzed the correlation between the risk groups derived from the comprehensive prognosis model and the clinical related information of patients, and drew a heat map.

### Prediction of chemotherapeutic drug resistance using a comprehensive prognostic model

First, based on the online database CellMiner (https://discover.nci.nih.gov/cellminer), we analyzed the interrelation between the expression of ceRNAs and drug sensitivity (30). At the same time, the correlation between the risk score of the comprehensive prognosis model and the half maximal inhibitor concentration (IC50) of antitumor drugs was analyzed by using pRRophetic software package.

### Differential expression analysis, survival analysis and biological function enrichment analysis of lncRNA PVT1

The ggpubr software package was used to analyze the differential expression of lncRNA plasmacytoma variant translocation 1 (PVT1), and further found out the correlation between lncRNA PVT1 and the clinical features of patients with GC. Then, the K-M survival curve of PVT1 was drawn by survival package and survivminer package. Finally, Gene Set Enrichment Analysis (GSEA), GO and KEGG enrichment analysis were used to explore the biological function of PVT1. The thresholds of the former were Nominal *p*-value < 0.05 and FDR Q-value < 0.25, and the thresholds of the latter two were *P* < 0.05.

### Validation of VCAN and RCAN2 expression and cellular localization in an integrated prognostic model

We utilized the HPA database (https://www.proteinatlas.org/). The expression levels of VCAN and RCAN2 in gastric cancer versus normal tissues were validated, and the cellular localization of both was further determined.

### Statistical analysis

All statistical analysis used R software 4.0.5 (Institute for Statistics and Mathematics, Vienna, Austria; www.r-project.org). All GC expression data downloaded from TCGA were in kilobase of exon per million reads mapped (FPKM) format. All data downloaded by GEO has been homogenized. The following R software packages were used for further data analysis: limma, glmnet, survival, timeROC, rms, ggpubr, survminer, pRRophetic. *P* < 0.05, the difference was statistically significant. When constructing the prognosis model, this study used a large sample size dataset, including more than 400 samples in the TCGA dataset and 300 and 192 samples in the two validation sets. The sample size fully met the requirements of subsequent studies. When there is an adjust P-value in all analyses, the threshold should not only be *P* < 0.05, but also adjust *P*-value < 0.05.

## Results

### Differential expression results

**Figure 1:**
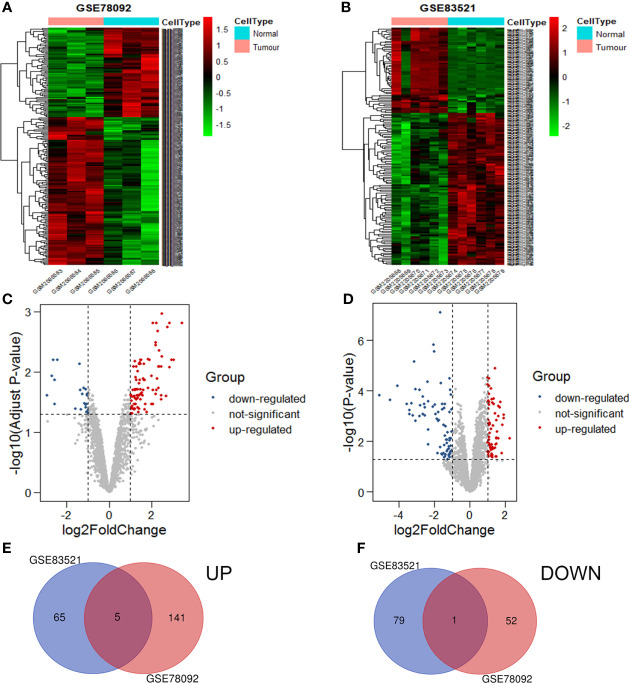
Volcanic maps and heat maps of circRNAs expression. The differential expression heat maps of GSE78092 **(A)** and GSE83521 **(B)**. The differential expression volcanic maps of GSE78092 **(C)** and GSE83521 **(D)**. Highly expressed circRNAs in the intersection of GSE78092 and GSE83521 **(E)**. Low expression circRNAs in the intersection of GSE78092 and GSE83521 **(F)**.

The circRNA expression profiles of GSE78092 and GSE83521 were used for differential expression analysis. As a result, 199 (146 up-regulated, 53 down-regulated) and 150 (70 up-regulated, 80 down-regulated) differentially expressed circRNAs were obtained **(** **Figures 1A** **–D** **)**. The intersection of the above two differentially expressed circRNAs was taken to obtain 6 final circRNAs (up-regulated 5, down-regulated 1) **(** **Figures 1E** **, F**; **Table 1** **)**. The results of differential expression analysis between TCGA and the corresponding GEO database were intersected to obtain miRNAs and mRNAs for further analysis.

**Table 1:** Details of circRNAs.

circRNA ID	Position	Strand	Best transcript	Gene symbol	Regulation
hsa_circ_0074854	chr5: 162940560-162944680	+	NM_182796	MAT2B	up-regulation
hsa_circ_0013048	chr1: 82302569-82372915	+	NM_012302	LPHN2	up-regulation
hsa_circ_0050102	chr19: 18459757-18466821	+	NM_017712	PGPEP1	down-regulation
hsa_circ_0000673	chr16: 11940357-11940700	–	NM_015659	RSL1D1	up-regulation
hsa_circ_0001658	chr6: 157357968-157406039	+	NM_017519	ARID1B	up-regulation
hsa_circ_0009172	chr10: 70218860-70229920	–	NM_001080449	DNA2	up-regulation

### Construction of a ceRNA regulatory network

**Figure 2:**
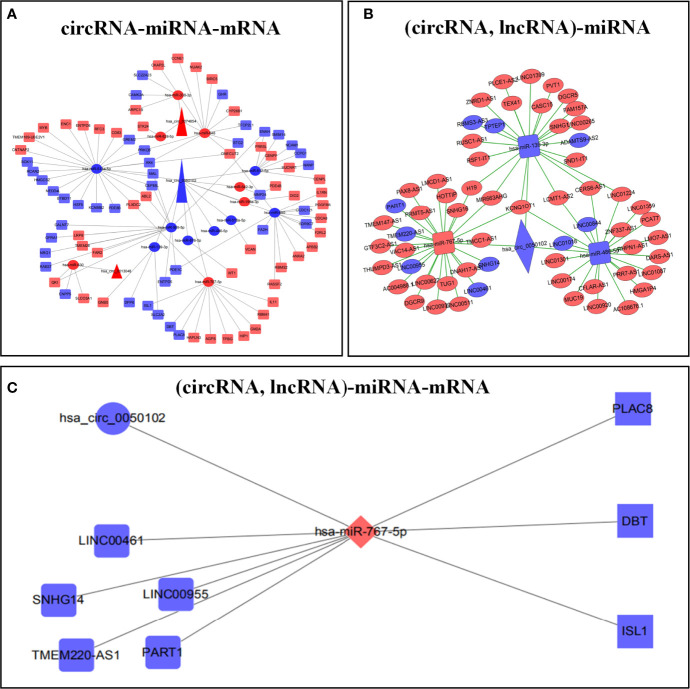
Construction of ceRNA prognostic models. circRNA-miRNA-mRNA regulatory network **(A)**. (circRNA, lncRNA)-mRNA regulatory network **(B)**. (circRNA, lncRNA)-miRNA-mRNA regulatory network **(C)**. Red represents up regulation, blue represents down regulation.

Circbank, Target Scan Human 7.2, miRWalk, miRDB, and LNCBaseV.2 databases were used to predict the corresponding ceRNAs. The predicted results were crossed with differentially expressed RNAs to construct the ceRNA regulatory network. We respectively constructed circRNA-miRNA-mRNA and (circRNA, lncRNA)-miRNA regulatory networks **(** **Figures 2A** **, B**; **Supplementary Tables 1**, **2** **)**. Finally, the two ceRNA networks were integrated to construct the (circRNA, lncRNA)-miRNA -mRNA regulatory network **(** **Figure 2C** **)**.

### Biological function annotation of the ceRNA regulatory network

Go analysis showed that the biological processes of the ceRNA regulatory network mainly focused on sister chromatid adhesion, MAPK cascade signaling pathway, protein phosphorylation, and negative transcriptional regulation of RNA polymerase II promoter. In cells, it was mainly concentrated in chromosomes, centromeres, and intermediates. The molecular functions were Ras-guanylate exchange factor activity and transcription activity **(** **Supplementary Table 3**, **Supplementary Figure 1A** **)**. KEGG analysis showed that it was mainly involved in purine metabolism and the ERBB signaling pathway. According to the results of enrichment analysis, the ceRNA regulatory network mainly activated the Ras-Raf-MAPK signaling pathway through phosphorylation of the ERBB receptor to regulate the replication, transcription, and translation of genetic material in the nucleus, thereby regulating the proliferation, differentiation, and metastasis of GC cells **(** **Supplementary Table 3**, **Supplementary Figure 1B** **)**. The PPI network had 54 nodes and 54 edges. The five genes with the highest connectivity were obtained by the Degree algorithm as key genes, including CDCA8 (Degree=6, BIRC5 (Degree=6), CENPF (Degree=5), NCAM1 (Degree=4), and AK4 (Degree= 4) **(** **Supplementary Figure 1C** **)**.

### Construction and verification of ceRNA-related prognostic models

**Figure 3:**
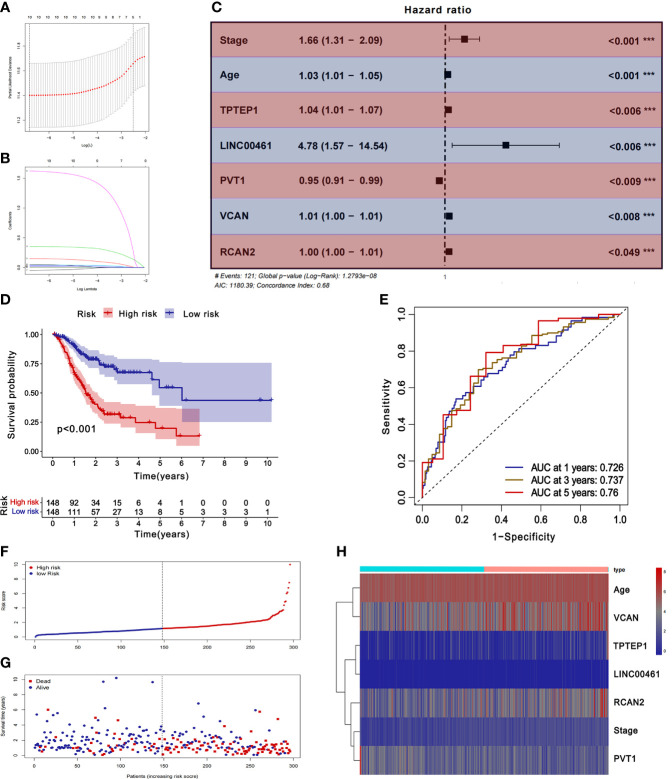
Construction and validation of ceRNA related prognostic model. **(A, B)** Lasso regression analysis was used to simplify the prognostic model. **(C)** Correlation between ceRNAs and prognosis. **(D)** K-M survival analysis of prognostic model. **(E)** ROC curve of prognostic model. **(F-H)** Survival status of gastric cancer patients with different risk scores.

The samples with missing RNA expression data and clinical data were excluded, and 296 patients with GC were finally included. The key prognostic biomarkers were obtained by univariate Cox regression analysis **(** **Supplementary Table 4** **)** and Lasso regression analysis **(** **Figures 3A** **, B** **)** and then the prognostic model was established by multivariate Cox regression analysis. The prognosis model included 2 mRNAs (VCAN, RCAN2), 3 lncRNAs (LINC00461, TPTEP1, PVT1), and 2 clinical features (stage, age) **(** **Figure 3C** **)**. The HR of all factors except PVT1 was greater than 1, indicating that PVT1 may be a protective factor for GC, and the rest were risk factors **(** **Supplementary Table 5** **)**. The samples were divided into the low-risk group (148 cases) and high-risk group (148 cases) based on the median of risk score. The results of K-M survival analysis of the model showed that the overall survival time of the high-risk group was significantly lower than that of the low-risk group (*P* < 0.001) **(** **Figure 3D** **)**. K-M survival analysis of all factors in the prognosis model found four meaningful prognostic markers (*P* < 0.05) **(** **Supplementary Figure 11** **)**. The receiver operating characteristic curve showed that the AUC values of 1-year, 3-year, and 5-year survival rates were 0.726, 0.737, and 0.76 respectively, suggesting that the predictive power of this prognostic model was moderate **(** **Figure 3E** **)**. **Figures 3F** **–H** showed the differences in the expression of survival status and prognostic markers in patients with GC at different risk scores.

### Correlation of the abundance of 22 infiltrating immune cells and immune/stromal scores with ceRNA prognostic models

The CIBERSORT algorithm was used to predict the composition of 22 infiltrating immune cells in GC. The results of the histogram **(** **Supplementary Figure 2A** **)**, and violin map **(** **Supplementary Figure 2B** **)** showed that there are 12 infiltrating immune cells with differential expression, including B cells naïve, B cells memory, Plasma cells, T cells CD8, T cells CD4 naïve, T cells gamma delta, Monocytes, Macrophages M0, Macrophages M1, Mast cells resting, Eosinophils, Neutrophils.

The ceRNA-related prognostic model was used to classify patients in high- and low-risk groups, and GSEA analysis was performed on the two groups. The results showed that the high-risk group was mainly enriched in transendothelial migration of leukocytes, TGF-β signaling pathway, chemokine signaling pathway, cytokine-receptor interaction, ECM-receptor interaction, etc. **(** **Figure 5A** **)**. Therefore, we speculate that the ceRNA-related prognosis model is related to the tumor immune microenvironment.

**Figure 4:**
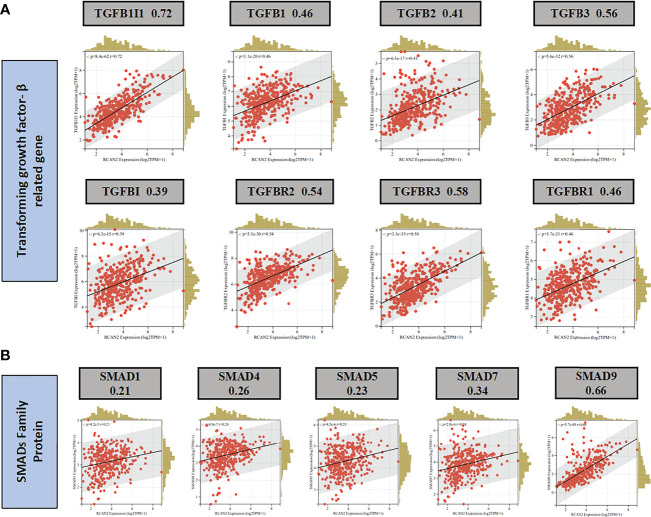
RCAN2 may adjust TGF- β signaling pathway. **(A)** RCAN2 was positively correlated with TGFB1-3 and TGFBR1-3 at the expression level. **(B)** RCAN2 was positively correlated with SMAD1,4,5,7,9 at the expression level.

**Figure 5:**
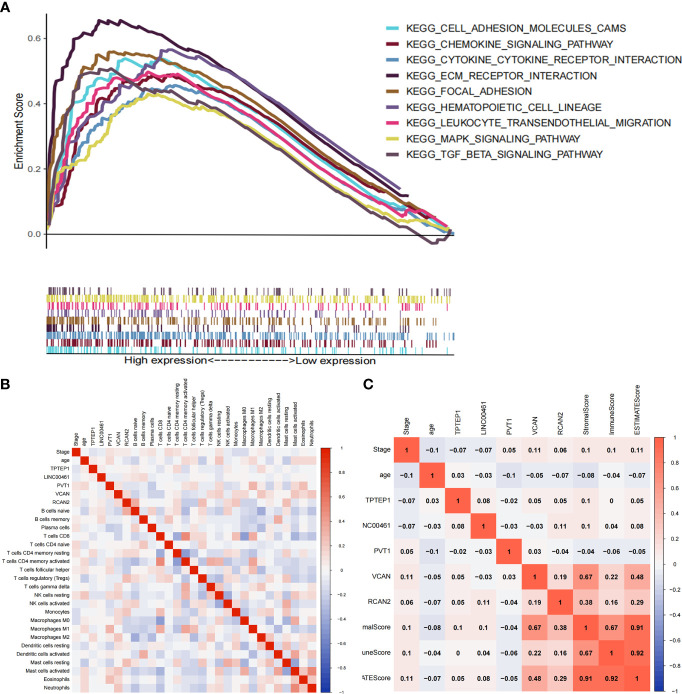
Correlation between ceRNAs and immune microenvironment of gastric cancer. **(A)** GSEA analysis of high-risk group divided by ceRNA related prognostic model. **(B)** Correlation analysis between ceRNAs and infiltrating immune cells. **(C)** Correlation analysis between ceRNAs and ImmuneScore/StromalScore.

Through GO enrichment analysis and KEGG enrichment analysis, we preliminarily speculated that RCAN2 may regulate the extracellular matrix through TGF-β pathway, thereby affecting the tumor extracellular immune microenvironment (**Supplementary Figure 3**). Then we showed that RCAN2 was positively correlated with TGFB1, TGFB2, TGFB3, TGFBR1, TGFBR2, TGFBR3, and TGFB1I1 in TGF-β pathway through correlation analysis (**Figure 4A**). In addition, RCAN2 was positively correlated with its downstream SMAD1, SMAD4, SMAD7, and SMAD9 at the expression level (**Figure 4B**). Finally, we verified that TGFB1I1, TGFBR1, SMAD9 and SMAD4 are low expressed in gastric cancer by immunohistochemistry at the protein level, and RCAN2 is also low expressed in gastric cancer (**Supplementary Figure 4**). It is preliminarily proved that RCAN2 can regulate TGF-βpathway, thereby affecting tumor immune microenvironment. At the same time, the immune/stromal score was obtained by using the ESTIMATE algorithm. Correlation analysis showed that there was a correlation between RNAs in the model and immune cell abundance. B cells naive-RCAN2, Mast cells resting-RCAN2, T cells regulatory-RCAN2, and Macrophages M1-PVT1 were all positively correlated. ESTIMATEScore-RCAN2, StromalScore-RCAN2, ImmuneScore-VCAN, StromalScore-VCAN, ESTIMATEScore-VCAN were all positively correlated **(** **Supplementary Figure 5A1–4** **, B1-5**; **Figures 5B** **, C** **)**. To sum up, RCAN2 may regulate the tumor invasion abundance of T cells regulation and mast cells through TGF-β signal pathway, and further regulate the tumor immune microenvironment.

**Figure 6:**
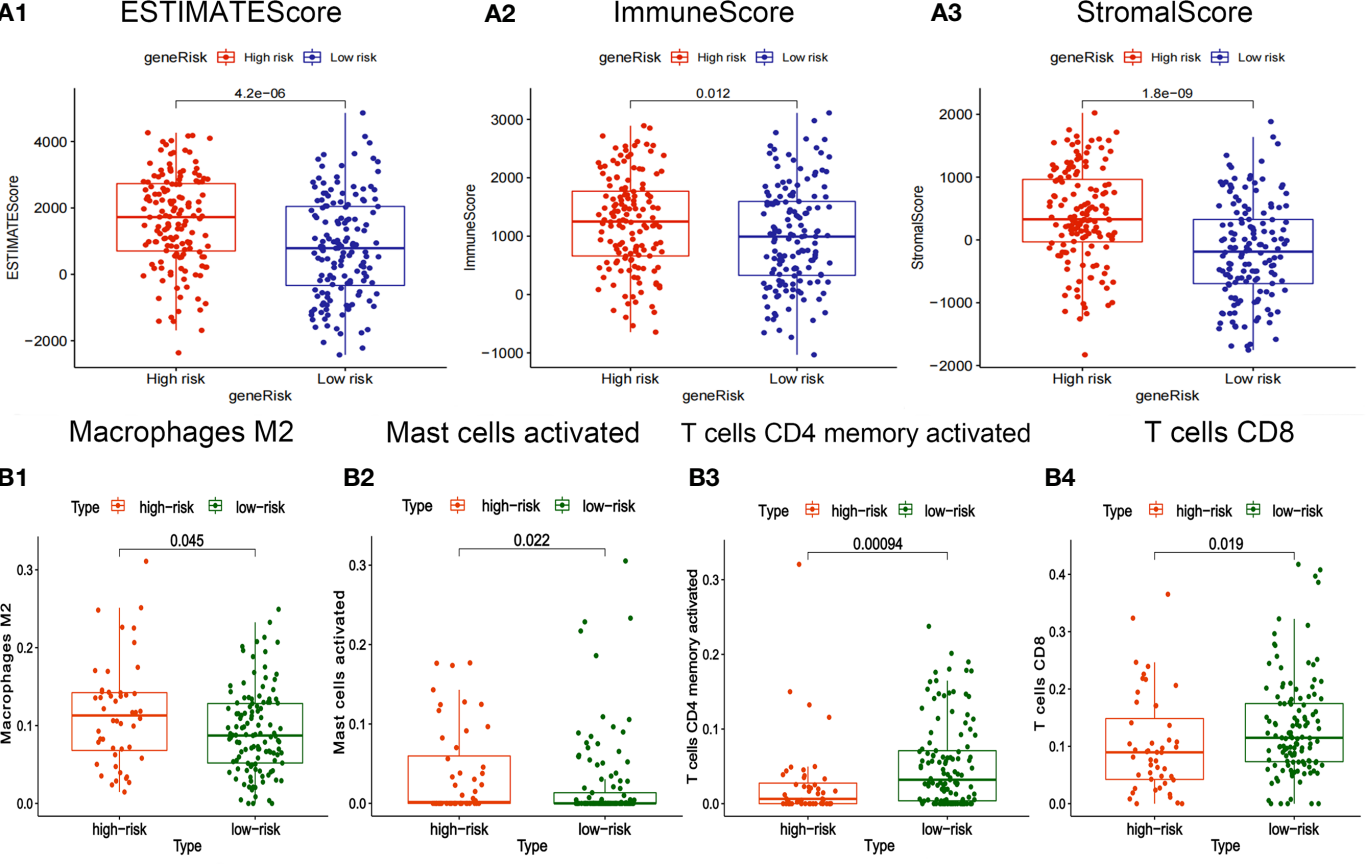
Correlation between risk-score of ceRNA related prognosis model and immune microenvironment. **(A1-3)** Correlation analysis between risk score and ImmuneScore/StromalScore of gastric cancer. **(B1-4)** Correlation analysis between risk-score and abundance of invasive immune cells in gastric cancer.

The box plot showed that the StromalScore, ImmuneScore, and ESTIMATEScore of the high-risk group are higher **(** **Figures 6** **A1–3** **)**. T cells CD8 and T cells CD4 memory activated have high infiltration abundance in the low-risk group, and Macrophages M2 and Mast cells activated had high infiltration abundance in the high-risk group **(** **Figures 6** **B1–4** **)**.

**Figure 7:**
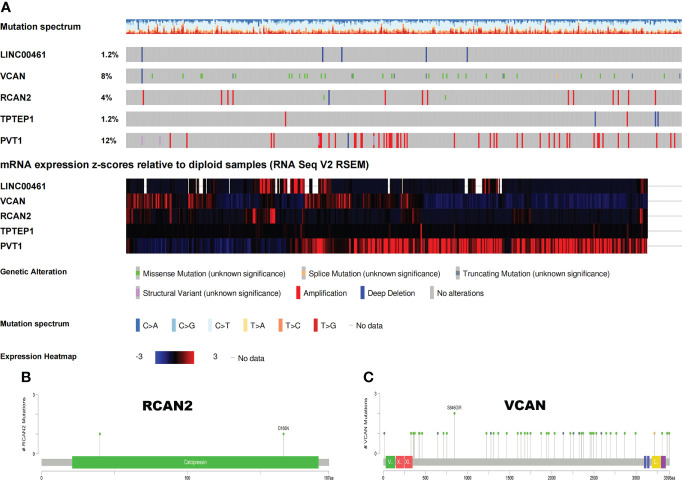
Somatic mutation map of 5 ceRNAs. **(A)** Mutation frequency and expression of 5 ceRNAs. **(B, C)** Mutation patterns of VCAN and RCAN2.

The somatic mutation profiles of 5 RNAs showed that the mutation frequencies of VCAN and PVT1 were high, 8% and 12%, respectively **(** **Figure 7A** **)**. The mutation patterns of VCAN and RCAN2 are shown in **Figures 7B** **, C**. Therefore, we speculate that the tumor mutation burden of RNAs in the ceRNA prognosis model is high, and the high tumor mutation burden can increase tumor heterogeneity and further lead to the change of tumor immune microenvironment.

### Construction of an immune-related prognosis model

22 kinds of immune cells, StromalScore, ImmuneScore, and ESTIMATEScore were used to construct the immune prognosis model. The immune prognosis model constructed by Lasso regression analysis **(** **Supplementary Figure 6A1-2** **)** and multivariate Cox regression analysis included 7 kinds of immune cells **(** **Supplementary Figure 6B**, **Supplementary Table 6** **)**. The K-M survival analysis of the model showed that patients with high-risk scores had a poorer prognosis **(** **Supplementary Figure 6C** **)**. The receiver characteristic curve showed that the AUC values of 1-year, 3-year, and 5-year survival rates are 0.7, 0.68, and 0.519 respectively, suggesting that the predictive ability of this prognosis model is mediocre and cannot be used for long-term prognosis prediction **(** **Supplementary Figure 6D** **)**. **Supplementary Figure 6E1–3** showed the differences in the expression of survival status and prognostic markers of GC patients with different risk scores. In addition, through K-M survival analysis, we found that Mast cells activated are a protective factor for GC, Mast cells resting is a risk factor for GC, and StromalScore is a protective factor for GC **(** **Supplementary Figure 11** **)**, which can be used for independent prognosis prediction.

### Construction and external validation of a comprehensive prognostic model combining ceRNAs and immunity

**Figure 8:**
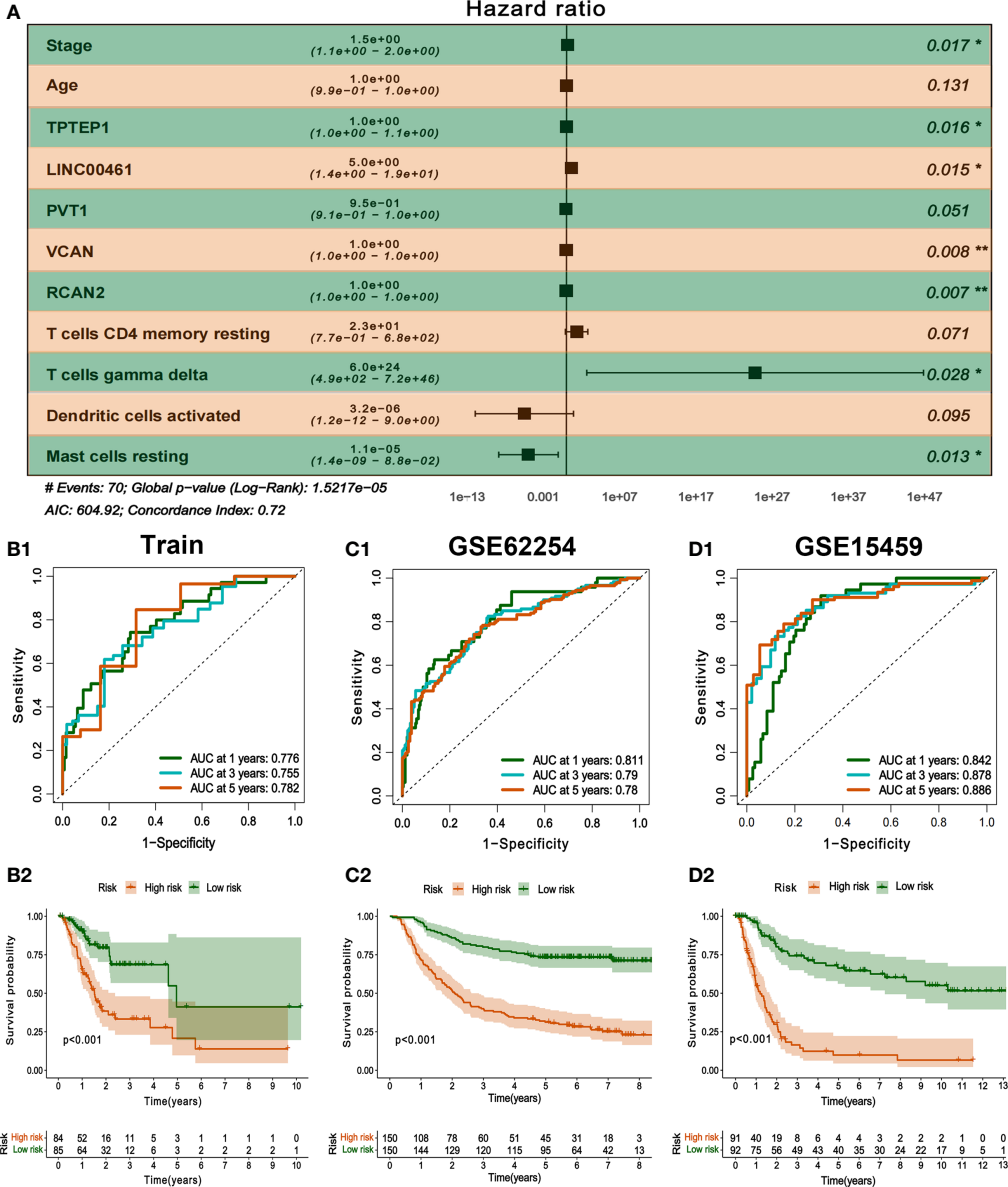
Construction and validation of comprehensive prognostic model. **(A)** Multivariate Cox model was used to construct a comprehensive prognosis model. **(B1-2)** ROC curve and K-M survival analysis of training set. **(C1-2, D1-2)** ROC curve and K-M survival analysis of GSE62254 and GSE15459 external validation set.

**Figure 9:**
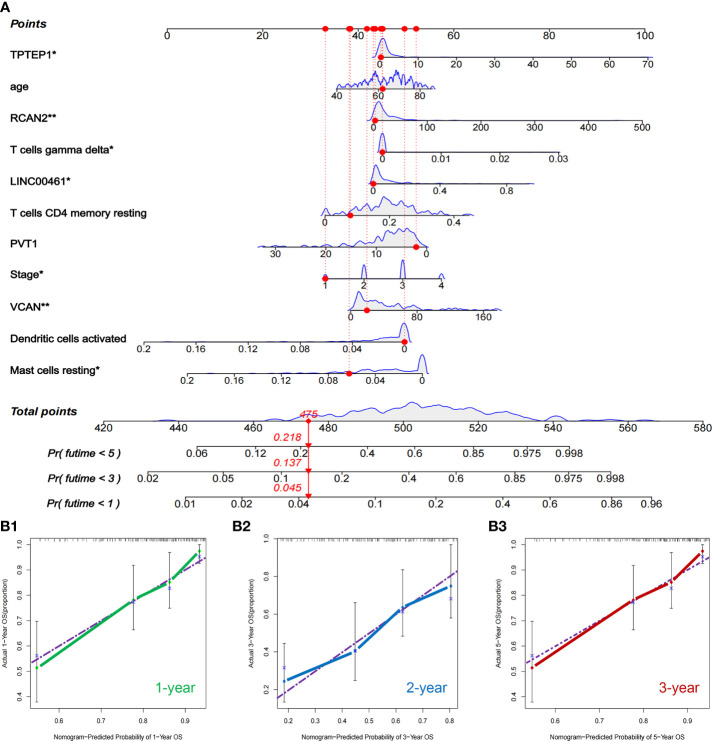
Nomogram and validation of comprehensive prognostic model. **(A)** Nomogram of the comprehensive prognostic model. **(B1–3)** The calibration curves for the nomogram.

All independent prognostic factors of ceRNAs related prognosis model and immune-related model (*P*-value of multivariate Cox analysis is less than 0.05) were integrated to construct a comprehensive prognosis model **(** **Supplementary Figure 7** **)**. The model included two clinical indicators (age and stage), three lncRNAs (TPTEP1, LINC00461, PVT1), two mRNAs (VCAN, RCAN2), and four infiltrating immune cells (T cells CD4 memory resting, T cells gamma delta, Dendritic cells activated, Mast cells resting) **(** **Figure 8A** **)**. Among them, the HR of PVT1, Dendritic cells activated, and Mast cells resting were less than 1, which was the protective factor of GC, and the other factors were the risk factors **(** **Table 2** **)**. The K-M survival analysis of the model showed that patients with high-risk score had a poorer prognosis (*P* < 0.001) **(** **Figure 8** **B2** **)**. The receiver characteristic curve showed that the AUC values of 1-year, 3-year, and 5-year survival rates were 0.776, 0.755, and 0.782 respectively, indicating that the predictive ability of the prognostic model was better **(** **Figure 8** **B1** **)**. The comprehensive prognosis model in the independent validation sets GSE62254 and GSE15459 was stable, and the K-M curve showed that the prognosis of patients with high-risk score was poor (*P* < 0.001) **(** **Figure 8** **C2** **, D2** **)**. In the validation set GSE62254, the AUC values of 1-year, 3-year, and 5-year survival rates were 0.811, 0.79, and 0.78, respectively **(** **Figure 8** **C1** **)**. In the validation set GSE15459, the AUC values of 1-year, 3-year, and 5-year survival rates were 0.842, 0.878, and 0.886, respectively **(** **Figure 8** **D1** **)**. It showed that the predictive ability of the comprehensive prognosis model was good and stable. **Supplementary Figure 8** showed the differences in the expression of survival status and prognostic markers of GC patients with different risk scores. The construction of the nomogram visualized all factors in the comprehensive model and the calibration curves of 1, 3, and 5 years showed that the prediction of the model was more consistent with the actual situation **(** **Figures 9A** **, B1–3** **)**. **Supplementary Figures 9** and **10** showed the nomograms of the comprehensive prognostic model in the external independent validation sets GSE62254 and GSE15459, respectively.

**Table 2:** Multivariate Cox regression analysis of comprehensive prognostic model.

ID	coef	HR	HR.95L	HR.95H	P value
Stage	0.375595	1.455857	1.068968	1.982773	**0.017**
age	0.020179	1.020384	0.994022	1.047445	0.131
TPTEP1	0.037176	1.037876	1.006875	1.069831	**0.016**
LINC00461	1.618377	5.044895	1.371381	18.55863	**0.015**
PVT1	-0.04893	0.952247	0.90658	1.000215	0.051
VCAN	0.008053	1.008085	1.002099	1.014107	**0.008**
RCAN2	0.005219	1.005233	1.001437	1.009042	**0.007**
T cells CD4 memory resting	3.128088	22.83028	0.767762	678.8844	0.071
T cells gamma delta	57.04703	5.96E+24	490.604	7.24E+46	**0.028**
Dendritic cells activated	-12.6463	3.22E-06	1.15E-12	9.003185	0.095
Mast cells resting	-11.4065	1.11E-05	1.40E-09	0.08806	**0.013**

Bold indicates that the result is meaningful.

**Figure 10:**
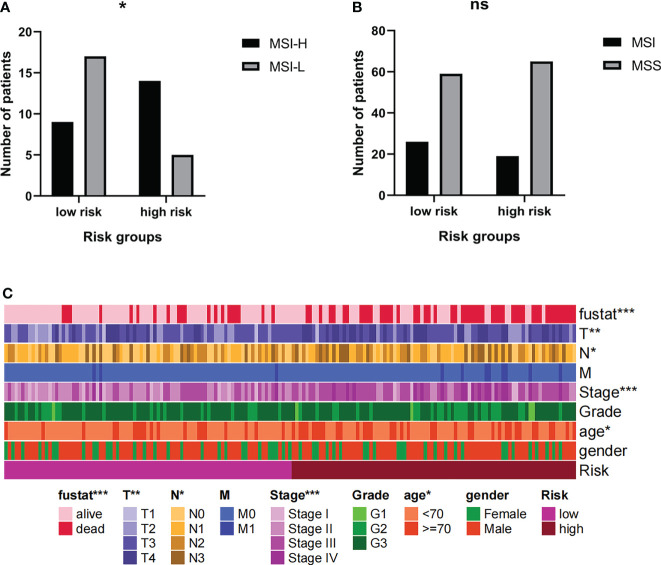
**(A, B)** The MSI levels of patients in high-risk groups were different. **(C)** Risk grouping is related to clinical indicators of patients. * Indicates P < 0.05. ** Indicates P < 0.01. *** Indicates P < 0.001. Ns indicates not statistically significant.

Current research shows that MSI-H/MSI-L has different responses to tumor immunotherapy, so we choose to further group MSI for analysis. The results showed that among MSI patients, the proportion of MSI-H patients in the high-risk group was higher (**Figure 10A**). However, the risk group cannot distinguish between MSI and MSS (**Figure 10B**). It can be inferred that the prognosis of patients in high-risk groups is poor, but immunotherapy may have a better effect.

The risk groups obtained from the comprehensive prognosis model are related to many clinical data. In short, being in the high-risk group means that the patient is more likely to be in a state of death, a higher T stage, a higher clinical stage, and an older age **(** **Figure 10C** **)**. This is consistent with clinical cognition.

### Prediction of chemosensitivity in patients with GC by a comprehensive prognostic model

**Figure 11:**
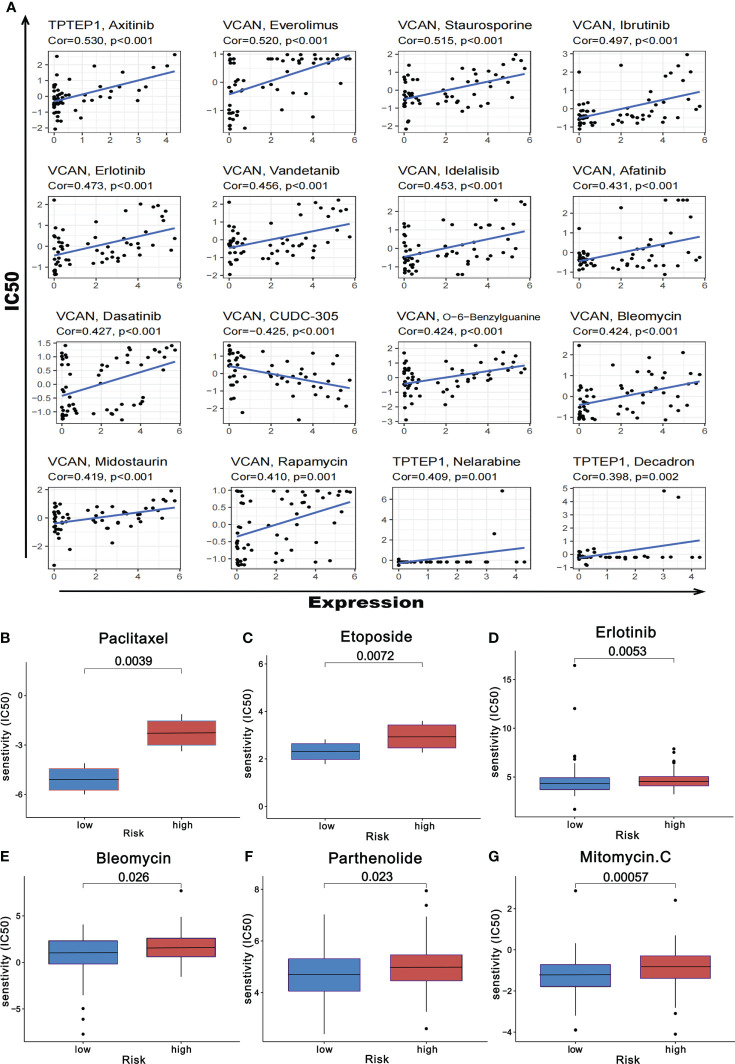
Prediction of chemotherapeutic drug resistance in gastric cancer by comprehensive prognosis model. **(A)** Correlation between five RNAs and chemotherapeutic drug resistance in gastric cancer. **(B-G)** The risk score obtained by the comprehensive model can predict the drug resistance of six common chemotherapeutic drugs.

Based on the online database CellMiner, the relationship between the 5 RNAs in the comprehensive model and chemotherapeutic drug sensitivity was obtained (*P* < 0.05). The figure showed some of the results with high correlation **(** **Figure 11A**, **Supplementary Table 7** **)**. In addition, according to the pRophetic algorithm, we predicted the IC50 of six commonly used chemotherapy drugs (paclitaxel, etoposide, bleomycin, parthenolide, mitomycin.C, and erlotinib) in patients with high-risk scores and low-risk scores (*P* < 0.05). It was found that all 6 drugs had higher IC50 in patients with high-risk scores **(** **Figures 11B** **–G** **)**, suggesting that patients with high-risk scores had poor sensitivity to the above drugs.

### The expression, prognosis prediction and biological function analysis of LncRNA PVT1

PVT1 may be a protective factor for GC, which we further analyzed (303 people remained after deleting the missing data). PVT1 was highly expressed in GC (*P* < 0.05) **(** **Supplementary Figure 12A, B** **)**. PVT1 was only associated with the T stage, and its expression was the highest in the T1 stage **(** **Supplementary Figure 12C** **)**. Taking the optimal expression level as the threshold, the patients with high expression of PVT1 (115 samples) and low expression (188 samples) were divided, and K-M survival analysis was performed. The results also showed that the patients with high expression of PVT1 had a better prognosis (**Supplementary Figure 12D**). The GSEA results showed that pathways such as base excision repair, cell cycle, DNA replication, homologous recombination, mismatch repair, nucleotide excision repair, and P53 signaling pathway were all enriched in the PVT1 high expression group, suggesting that high PVT1 expression was related to DNA repair **(** **Supplementary Figure 12E** ; **Supplementary Table 8** **)**. Pathways such as arrhythmogenic right ventricular cardiomyopathy, calcium signaling pathway, dilated cardiomyopathy, hypertrophic cardiomyopathy, and long-term depression were all enriched in the PVT1 low expression group, suggesting that low PVT1 expression was related to heart disease and depression **(** **Supplementary Figure 12F** ; **Supplementary Table 9** **)**. Correlation analysis showed that the expression of PVT1 was related to CLEC3B, ATAD2, DCAF13, and other genes (only the absolute value of correlation coefficient was greater than 0.4) **(** **Supplementary Figure 12G1–8** **)**. Further, GO and KEGG analysis showed that PVT1 may be related to DNA helicase, DNA replication, DNA repair, and cell cycle pathways **(** **Supplementary Figure 12H** **, I** ; **Supplementary Table 10** **)**. The correlation between PVT1 and the cell cycle pathway is shown in **Supplementary Figure 13**.

### Protein expression levels and cellular spatial localization of VCAN and RCAN2

**Figure 12:**
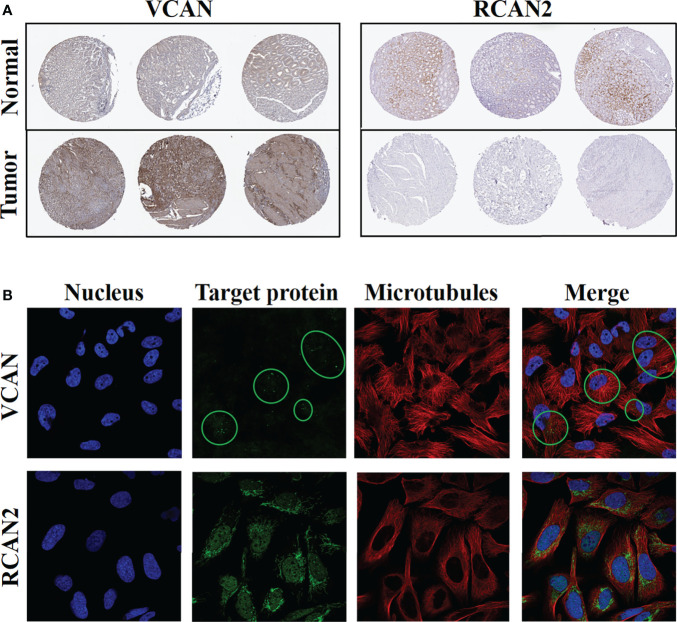
Protein expression levels and cellular spatial localization of VCAN and RCAN2. **(A)** Immunohistochemical experiments of VCAN and RCAN2 in gastric cancer and normal tissues. **(B)** The immunofluorescence experiments of HPA database showed that VCAN mainly exists in the vesicles of U-251 MG cells, and RCAN2 mainly exists in the mitochondria and nucleoplasm of U-2 OS cells.

Immunohistochemistry confirmed the above-mentioned high expression of VCAN in gastric cancer and low expression of RCAN2 in gastric cancer **(** **Figure 12A** **)**. Immunofluorescence experiments **(** **Figure 12B** **)** showed that VCAN mainly existed in vesicles in U-251 MG cells, which indicated that VCAN might be a secreted protein, which was most used outside the cell. While RCAN2 was detected in mitochondria and nucleoplasm in U-2 OS cells, suggesting that RCAN2 may be an intracellular protein.

## Discussion

Currently, there have been many studies focusing on lncRNA-miRNA-mRNA regulatory networks in GC. For example, researchers have found that lncRNA UBE2CP3 promotes the progression of GC by regulating the miR-138-5p/ITGA2 axis (31), and LncRNA NKX2-1-AS1 activates VEGFR-2 signal pathway by regulating miR-145-5p/SERPINE1 axis, which promotes angiogenesis and GC progression (32). In addition, a growing number of studies have begun to note that circRNAs and the circRNA-miRNA-mRNA ceRNA regulatory network also play an important role in GC. Researchers found that circMAPK1 can inhibit the phosphation of MAPK1 *via* the encoded protein MAPK1-109aa, so as to inhibit the procession of GC (33). circRNA_ 100290 promotes the proliferation and metastasis of GC cells by regulating the miR-29b-3p/ITGA11 axis (34). circCUL2 promotes the apoptosis of GC cells by regulating the miR-142-3p/ROCK2 axis, inhibits the procession of GC, and improves the cisplatin resistance of GC cells (35). It can be seen from above that both LncRNAs and circRNAs can act as molecular sponges and further regulate the process of GC by adsorbing miRNAs.

However, so far, few researchers have combined LncRNAs and circRNAs to construct a ceRNA regulatory network to further explore the molecular mechanism of GC. In this study, we predicted the targeted regulation relationship of RNA-RNA through multiple online databases, constructed two ceRNA regulatory networks, circRNA-miRNA-mRNA and (circRNA, lncRNA)-miRNA, and further combined these two networks to construct the (circRNA, lncRNA)-miRNA-mRNA ceRNA regulatory network. According to the results of enrichment analysis, the ceRNA regulatory network mainly regulated the replication, transcription, and translation of genetic material in the nucleus by regulating ERBB receptor phosphorylation and activating Ras-Raf-MAPK signaling pathway, to regulate the proliferation, differentiation, and metastasis of GC cells. We believed that the (circRNA, lncRNA)-miRNA-mRNA regulatory network can better conform to the molecular mechanism of ceRNA in GC, which was conducive to further exploring new therapeutic targets and prognostic markers. Furthermore, we constructed a ceRNA-related prognostic model by Cox regression analysis and Lasso regression analysis. The receiver characteristic curve of the model showed that the AUC values of 1-year, 3-year, and 5-year survival rates were 0.726, 0.737, and 0.76 respectively, indicating that the predictive ability of the prognostic model was medium. In addition, K-M survival analysis suggested that age, Stage, PVT1, and VCAN could be regarded as independent prognostic markers for GC patients.

Besides, we found that LncRNA PVT1 was highly expressed in GC and correlated with the T stage. The K-M survival analysis showed that high expression of PVT1 was a protective factor for GC. The results of enrichment analysis showed that the high expression of PVT1 was related to DNA repair (base precision repair, cell cycle, DNA replication, homologous recombination, mismatch repair, nucleoside precision repair, p53 signaling pathway, etc.); Low expression of PVT1 was associated with heart disease and depression (arrhythmogenic right ventricular cardiology pathway, calcium signaling pathway, divided cardiology pathway, hypertrophic cardiology pathway, long term depression, etc.). The results of enrichment analysis also supported that the high expression of PVT1 may be a protective factor for GC. However, many current studies have found that high expression of PVT1 can promote the proliferation, invasion, and metastasis of GC cells (36), promote the formation of neovascularization (37), and epithelial-mesenchymal transition (38), and indicate a poor prognosis (39). For the above inconsistent results, we believe that the molecular mechanism of GC involved in PVT1 and its relationship with the prognosis of GC patients still need further in-depth research. We can start with the results of enrichment analysis in this paper, such as further research on the correlation between PVT1 and DNA repair.

This study showed that the abundance of infiltrating immune cells is related to the progression and prognosis of GC. In addition, there is also evidence that immune/stromal score is an important influencing factor of GC (40, 41). According to CIBERSORT and ESTIMATE algorithms, we predicted the abundance of infiltrating immune cells and immune/stromal score in patients with GC. Based on this, an immune-related prognosis model was constructed. The receiver characteristic curve showed that the AUC values of 1-year, 3-year, and 5-year survival rates were 0.7, 0.68, and 0.519 respectively, suggesting that the predictive ability of the prognosis model was average. In addition, we found a very interesting independent prognostic factor: mast cells. A high proportion of activated mast cells indicates a poor prognosis, while a high proportion of resting mast cells indicates a good prognosis. These results suggest that inhibition of mast cells activity in the tumor microenvironment may be a new target for the GC treatment, which has also been preliminarily confirmed by researchers.

T cells gamma delta (γδ T cells) are unique subsets of T cells, which are not restricted by MHC in recognizing tumor antigens. So, they are defined as innate immune cells. γδ T cells can be divided into different groups according to the expression of γ chain or δ chain (42). The most studied of which are the Vδ1 T cells distributed in tissues and the Vγ9Vδ2 T cells distributed in peripheral blood. Peripheral Vδ1 T cells and Vγ9Vδ2 T cells could recognize tumor cells through TCRγδ and NKR, and kill them through perforin-granzyme B, Fas/FasL and TRAIL. Activated Vγ9Vδ2 T cells could perform the function of APC, and furthermore, they could activate NK cells and DC directly (43). On the contrary, tumor-infiltrating Vδ1 T cells promoted tumor development by secreting IL-17 and inhibiting the maturation of CD4/CD8 T cells and DC (44). γδ T cells have been used in clinical anti-tumor therapy and have achieved good results. The most common approach is to directly activate the antitumor activity of Vγ9Vδ2 T cells, either by *in vitro* stimulation or *in vivo*, and then apply them in tumor patients *via* different pathways (43). It is noteworthy to mention that although Vδ1 T cells account for the majority of tumor-infiltrating γδ T cells, the definition of γδ T cell subsets still rely on their profile in cytokine production. In the present study tumor infiltrating γδ T cells are an independent prognostic factor in gastric cancer, and their high infiltration level is closely associated with poor prognosis. This is also in keeping with the above studies.

In gastric cancer (45), thyroid cancer (46), pancreatic cancer (47), mast cells often display a pro tumorigenic effect and high infiltration levels of mast cells often predict poor patient outcome. However, it was found in breast cancer that the infiltration of mast cells can exert antitumor effects, and their infiltration tends to be beneficial to the prognosis of patients (48). While in melanoma, the contribution of mast cells to their development is not well defined and its role depends on the infiltration site of mast cells as well as on the subtype of the tumor (49, 50). This seemingly contradictory result illustrates that mast cells and their mediators have complex roles in different types of tumors.

Studies have shown that ceRNA regulatory network is related to the tumor immune microenvironment (51), and various RNAs are also related to tumor immunity (52–54). Through the correlation analysis of the two, we found similar conclusions. B cells naive-RCAN2, Mast cells resting-RCAN2, T cells regulatory-RCAN2, Macrophages M1-PVT1 are all positively correlated. ESTIMATEScore-RCAN2, StromalScore-RCAN2, ImmuneScore-VCAN, StromalScore-VCAN, ESTIMATEScore-VCAN are all positively correlated. We used KEGG enrichment analysis to predict that RCAN2 can regulate TGF signal pathway, thereby changing the immune cell infiltration in the tumor microenvironment (including B cells naive, mast cells, T cells regulatory), thereby changing the tumor immune situation. The somatic mutation map showed that the mutation evaluation rates of ceRNAs are relatively high, and the mutation frequencies of VCAN and PVT1 were 8% and 12%, respectively. Therefore, we speculate that the high mutation frequency of ceRNAs leads to greater tumor heterogeneity and changes in the immune status in the tumor microenvironment. However, it is very difficult to directly measure the abundance of infiltrating immune cells in GC, which also costs more money and time. Through this study, we have found a method to indirectly determine the proportion of immune cell infiltration, and predict the abundance of a certain infiltrating immune cell by measuring the expression of a certain RNA. This is a simple and quick idea, which provides a breakthrough for the study of the immune microenvironment of GC.

In addition, we found that the risk score calculated by the ceRNA-related prognosis model was related to the abundance of infiltrating immune cells and immune/stromal scores of GC, which implied that there may be a correlation between the ceRNA-related prognosis model and immune-related prognosis model. Therefore, we integrated the two models to build a new comprehensive model. The receiver characteristic curve showed that the AUC values of 1-year, 3-year, and 5-year survival rates were 0.776, 0.755, and 0.782 respectively. In the two external validation sets, the performance of the model was more brilliant, which indicated that the prediction ability of the prognosis model was excellent and pretty stable. At the same time, we assessed the correlation between the comprehensive prognosis model and the chemotherapeutic drug resistance of GC. The results showed that, on the one hand, the RNAs in the model could predict the sensitivity of chemotherapeutic drugs alone. On the other hand, the IC50 of six common chemotherapeutic drugs (paclitaxel, etoposide, bleomycin, parthenolide, mitomycin.C, and erlotinib) were higher in patients with high-risk scores, suggesting that patients with high-risk scores had poor sensitivity to the above drugs. We believe that the construction of a comprehensive prognostic model further improves the current situation of insufficient prognostic evaluation ability of clinical indicators, and is a powerful tool for prognostic evaluation of patients with GC. At the same time, the prognosis model can also predict the drug resistance of chemotherapy patients, which undoubtedly provides a novel idea for the majority of GC patients to select chemotherapy drugs.

At present, there are many prognostic models based on gastric cancer, but some of them do not have good predictive ability. Or it only performs well in the training set but performs poorly in the verification set, that is, the model does not have universal applicability. For example, the prognostic model of Huo et al. (55) contains 11 genes, which perform well in the training set, but perform poorly in multiple GEO database validation sets. However, the four gene prognostic model of Jia et al. (56) and Guo et al. (57) showed poor performance in TCGA training set and multiple GEO validation sets. We believe that our prognosis model has the following advantages: 1. Our comprehensive prognosis model performs well in TCGA training set and two GEO validation sets, which shows that the accuracy and stability of our prognostic model are satisfactory. 2. This model can not only predict the prognosis, but also predict the drug resistance of gastric cancer patients, which is conducive to clinical selection of more sensitive individualized chemotherapy programs. This prognostic model consists of clinical features, ceRNAs and a variety of immune infiltrating cells. It is a bold innovation and has achieved satisfactory success in the end to infer the prognosis of patients from multiple perspectives during the development of gastric cancer. However, this combined prognosis model is too complex. It needs to determine the expression level of each component in the model through transcriptome gene sequencing, further determine the risk score of patients and then infer their prognosis. However, the high price of gene chips has made it impossible to be widely used in clinical applications.

## Conclusion

In summary, we built two ceRNA regulatory networks, circRNA-miRNA-mRNA and (circRNA, lncRNA)-miRNA based on circRNAs. Then we integrated them into a (circRNA, lncRNA)-miRNA-mRNA regulatory network which is conducive to further improving the molecular mechanism of ceRNA in GC. At the same time, we constructed two prognostic models based on ceRNAs and immunity in tumor microenvironment, and further integrated the two to develop a comprehensive prognostic model. The model was a fully developed and reliable GC prognostic model with outstanding performance in TCGA training set and two GEO verification sets. In addition, we found that the abundance of infiltrating immune cells in GC was associated with ceRNAs, so we could predict the abundance of infiltrating immune cells by the relevant RNAs expression, which is a very simple method. Excitingly, we found two interesting independent prognostic markers: LncRNA PVT1 and mast cells. In this paper, LncRNA PVT1 is a protective factor for GC, but there is a contradiction with related literature. It’s our next stage of work to consider whether PVT1 can participate in the process of GC through the gene repair pathway. The activation of mast cells is a risk factor for GC. Therefore, the inhibition or therapeutic depletion of mast cells in the tumor microenvironment is a promising new treatment option for GC.

## Data availability statement

The original contributions presented in the study are included in the article/**Supplementary Material**. Further inquiries can be directed to the corresponding authors.

## Author contributions

Conceptualization: WC and GC. Methodology: WC, GC, and ZZ. Software: WC, WZ, SY, and ZZ. Formal analysis: WC, ML, and KY. Investigation: WC and SY. Data curation: WC and WZ. Project administration: BC and MX. Writing–original draft preparation: WC and ML. Writing–review and editing: WC, ML, WZ, and XZ. Visualization: WC and KY. Funding acquisition: BC and MX. All authors contributed to the article and approved the submitted version.

## Funding

This work was supported by the National Natural Science Foundation of China (81602425), Anhui Provincial Natural Science Foundation (2208085MH240), the Anhui Quality Engineering Project (2020jyxm0898, 2020jyxm0910), the Anhui Health Soft Science Research Project (2020WR01003), and the Key Research and Development Program of Anhui Province (201904a07020045).

## Conflict of interest

The authors declare that the research was conducted in the absence of any commercial or financial relationships that could be construed as a potential conflict of interest.

## Publisher’s note

All claims expressed in this article are solely those of the authors and do not necessarily represent those of their affiliated organizations, or those of the publisher, the editors and the reviewers. Any product that may be evaluated in this article, or claim that may be made by its manufacturer, is not guaranteed or endorsed by the publisher.

## Supplementary material

Supplementary Figure 1 Enrichment analysis of mRNA and construction of PPI network. **(A)** The GO analysis of mRNA. **(B)** The KEGG analysis of mRNA. **(C)** The protein- protein interaction network of mRNA. Red represents up regulation, and blue represents down regulation, and ellipses represent key genes.

Supplementary Figure 2 CIBERSORT algorithm was used to predict the composition of 22 invasive immune cells in gastric cancer. **(A)** Abundance of 22 immune cells in gastric cancer. Violin picture **(B)** of the proportion of 22 immune cells in gastric cancer.

Supplementary Figure 3 RCAN2 **(A)** KEGG enrichment analysis. **(B)**. GO enrichment analysis.

Supplementary Figure 4 The expression of TGF- β pathway related proteins related to RCAN2 in tumor and normal tissues.

Supplementary Figure 5 Correlation between ceRNAs **(A1-4)** and immune microenvironment **(B1-3)** of gastric cancer.

Supplementary Figure 6 Construction and validation of immune related diagnostic model. **(A1-2)** Lasso regression analysis was used to simplify the prognostic model. **(B)** Correlation between infiltrating immune cells and diagnosis **(C)** K-M survival analysis of prognostic model. **(D)** ROC curve of prognostic model. **(E1-3)** Survival status of gastric cancer patients with different risk scores.

Supplementary Figure 7 Lasso regression analysis. **(A)** Use the minimum criterion of 10 times cross validation to select adjustment parameters in lasso regression. **(B)** Characteristic curve of lasso coefficient.

Supplementary Figure 8 The differences in the expression of survival status and prognostic markers of GC patients with different risk scores. **(A1-3)** Training set based on TCGA database. **(B1-3)** Verification set based on GEO62254. **(C1-3)** Verification set based on GEO15459.

Supplementary Figure 9 The nomogram of the comprehensive prognostic model in the external independent validation set GSE62254.

Supplementary Figure 10 The nomogram of the comprehensive prognostic model in the external independent validation set GSE15459.

Supplementary Figure 11 Independent prognostic biomarkers of gastric cancer. **(A)** age, **(B)** Stage, **(C)** PVT1, **(D)** VCAN, **(E)** Mast cells activated, **(F)** Mast cells resting, **(G)** StromalScore.

Supplementary Figure 12 Expression, prognosis and biological function analysis of LncRNA PVT1. **(A, B)** PVT1 is highly expressed in gastric cancer. **(C)** PVT1 is associated with T-stage of gastric cancer. **(D)** K-M curve showed that PVT1 was a protective factor for gastric cancer. **(E)** GSEA analysis of patients with high PVT1 expression. **(F)** GSEA analysis of patients with low PVT1 expression. **(G1-8)** Correlation between PVT1 and genes. **(H, I)** Go and KEGG enrichment analysis of PVT1.

Supplementary Figure 13 Correlation between PVT1 and cell cycle pathway.
