# Supplementary material for: A novel signature based on CeRNA and immune status predicts prognostic risk and drug sensitivity in gastric cancer patients
Source: Front Immunol. 2022 Nov 22;13:951135. doi: 10.3389/fimmu.2022.951135 (PMC9723231; doi:10.3389/fimmu.2022.951135)
Supplement: Supplementary file 1 [file DataSheet_1.docx]

**Supplementary Table 1** CeRNA regulatory network of circRNA-miRNA-mRNA

| circRNA | miRNA | mRNA | | | | | | |
| --- | --- | --- | --- | --- | --- | --- | --- | --- |
| hsa_circ_0074854 | hsa-miR-646 | BIRC5 | CCNE1 | CYP26B1 | CKAP2L | NUAK2 | ONECUT2 |  |
|  |  | BTG2 | PRKCB | AK4 | GHR | TFCP2L1 |  |  |
|  | hsa-miR-338-3p | ARPC1B | SLC22A23 | CAMK2A |  |  |  |  |
|  | hsa-miR-628-5p | STK24 | GREM2 |  |  |  |  |  |
| hsa_circ_0013048 | hsa-miR-630 | SLCO3A1 | QKI | ENPP5 |  |  |  |  |
| hsa_circ_0050102 | hsa-miR-513a-5p | RFC3 | ABL2 | CD83 | ENTPD6 | PLXDC2 | CNTNAP2 |  |
|  |  | TMEM189-UBE2V1 | ENC1 | ONECUT2 | MYB | GREM2 | HMGCS2 |  |
|  |  | PRKCB | AK4 | CEP85L | BTBD11 | RCAN2 | MAL |  |
|  |  | NEDD4L | KCNMB2 | SOX11 | PDE8B | HSF5 |  |  |
|  | hsa-miR-650 | APBB2 | RBMS2 | PDGFRB | ANXA2 | F2RL2 | RASSF2 |  |
|  |  | IL1RN | ONECUT2 | CDCA8 | PDE1C | SDR9C7 | FA2H | CCDC121 |
|  | hsa-miR-139-3p | GNB5 | SLC2A2 | DPP6 |  |  |  |  |
|  | hsa-miR-486-5p | NA |  |  |  |  |  |  |
|  | hsa-miR-876-5p | WT1 |  |  |  |  |  |  |
|  | hsa-miR-516a-5p | VCAN |  |  |  |  |  |  |
|  | hsa-miR-509-5p | ABL2 | QKI | FAR2 | LRP8 | TMEM26 | PDE1C | AK4 |
|  |  | RAB37 | CEP85L | ENAH | GFRA1 | GALNT7 | NRG1 | ENTPD5 |
|  | hsa-miR-512-5p | CENPL | PRR5L | SUCNR1 | CENPF | CCPG1 | NCAM1 | SMIM14 |
|  |  | ENAH | NANP |  |  |  |  |  |
|  | hsa-miR-542-3p | MMP24 |  |  |  |  |  |  |
|  | hsa-miR-199a-3p | DIO2 | PDE4B |  |  |  |  |  |
|  | hsa-miR-767-5p | AGPS | TPBG | GM2A | PLXDC2 |  |  |  |
|  |  | IL11 | HIP1 | RBM41 | HAPLN3 | ISL1 | DBT | PLAC8 |

NA stands for no gene.

**Supplementary Table 2** CeRNA regulatory network of (circRNA, lncRNA)-miRNA

| circRNA | miRNA | lncRNA | | | |
| --- | --- | --- | --- | --- | --- |
| hsa_circ_0050102 | hsa-miR-767-5p | SNHG16 | GTF3C2-AS1 | KCNQ1OT1 | LINC00461 |
|  |  | AC004988.1 | MIR663AHG | H19 | LINC00955 |
|  |  | DNAH17-AS1 | THUMPD3-AS1 | TMEM147-AS1 | TMEM220-AS1 |
|  |  | PAX8-AS1 | LINC00939 | PRMT5-AS1 | SNHG14 |
|  |  | LMCD1-AS1 | TUG1 | HOTTIP |  |
|  |  | LINC00622 | DGCR9 | TMCC1-AS1 |  |
|  |  | VAC14-AS1 | LINC00511 | PART1 |  |
|  | hsa-miR-139-3p | RSF1-IT1 | SNHG1 | TEX41 | PLCE1-AS2 |
|  |  | LINC01399 | SND1-IT1 | LINC00265 | TPTEP1 |
|  |  | DGCR5 | MIR663AHG | LCMT1-AS2 | RBMS3-AS3 |
|  |  | FAM157A | CERS6-AS1 | RUSC1-AS1 | ADAMTS9-AS2 |
|  |  | CASC15 | KCNQ1OT1 | PVT1 |  |
|  |  | ZNRD1-AS1 | H19 | LINC01224 | LINC01359 |
|  | hsa-miR-486-5p | LINC01301 | AC108676.1 | DARS-AS1 | LINC00844 |
|  |  | RHPN1-AS1 | CERS6-AS1 | LINC01087 | LINC01018 |
|  |  | LINC00174 | KCNQ1OT1 | LINC00920 |  |
|  |  | CFLAR-AS1 | HMGA1P4 | PRR7-AS1 |  |
|  |  | PCAT7 | MUC19 | LCMT1-AS2 |  |
|  |  | LMO7-AS1 | ZNF337-AS1 | LINC01224 |  |

**Supplementary Table 3** Enrichment analysis of ceRNA network

| Term |  | Count | GeneRatio | P Value | Genes |
| --- | --- | --- | --- | --- | --- |
| BP |  |  |  |  |  |
| GO:0007062 | sister chromatid cohesion | 4 | 4.71% | 0.0122 | CENPF, CENPL, CDCA8, BIRC5 |
| GO:0006468 | protein phosphorylation | 7 | 8.24% | 0.0193 | RASSF2, NUAK2, STK24, CCNE1, PRKCB, CAMK2A, BIRC5 |
| GO:0000165 | MAPK cascade | 5 | 5.88% | 0.0337 | PDGFRB, CAMK2A, GFRA1, NRG1, NCAM1 |
| GO:0000122 | negative regulation of transcription from RNA polymerase II promoter | 8 | 9.41% | 0.0496 | BTG2, WT1, MYB, NEDD4L, SOX11, TFCP2L1, ISL1, LRP8 |
| CC |  |  |  |  |  |
| GO:0000775 | chromosome, centromeric region | 4 | 4.71% | 0.0022 | CENPF, CENPL, CDCA8, BIRC5 |
| GO:0030496 | midbody | 4 | 4.71% | 0.0205 | CENPF, ANXA2, CDCA8, BIRC5 |
| MF |  |  |  |  |  |
| GO:0005088 | Ras guanyl-nucleotide exchange factor activity | 5 | 5.88% | 0.0020 | PDGFRB, CAMK2A, GFRA1, NRG1, NCAM1 |
| GO:0004114 | 3',5'-cyclic-nucleotide phosphodiesterase activity | 3 | 3.53% | 0.0050 | PDE1C, PDE4B, PDE8B |
| GO:0001077 | transcriptional activator activity, RNA polymerase II core promoter proximal region sequence-specific binding | 5 | 5.88% | 0.0238 | ONECUT2, WT1, MYB, SOX11, ISL1 |
| KEGG PATHWAY |  |  |  |  |  |
| hsa05032 | Morphine addiction | 5 | 5.88% | 0.0020 | PDE1C, PRKCB, PDE4B, GNB5, PDE8B |
| hsa00230 | Purine metabolism | 6 | 7.06% | 0.0037 | PDE1C, ENTPD5, ENTPD6, PDE4B, AK4, PDE8B |
| hsa04012 | ErbB signaling pathway | 4 | 4.71% | 0.0147 | PRKCB, CAMK2A, ABL2, NRG1 |

**Supplementary Table 4** Univariate Cox regression analysis of ceRNAs

| id | HR | HR.95L | HR.95H | P value |
| --- | --- | --- | --- | --- |
| T | 1.277567 | 1.01396 | 1.609705 | 0.037751 |
| N | 1.289689 | 1.097568 | 1.515438 | 0.001994 |
| Stage | 1.476543 | 1.182952 | 1.842999 | 0.00057 |
| age | 1.020675 | 1.002616 | 1.039059 | 0.024653 |
| TPTEP1 | 1.03817 | 1.009994 | 1.067131 | 0.007622 |
| LINC00461 | 4.193859 | 1.390333 | 12.65053 | 0.010929 |
| PVT1 | 0.958375 | 0.922727 | 0.995401 | 0.027927 |
| VCAN | 1.005873 | 1.001521 | 1.010244 | 0.008123 |
| RCAN2 | 1.004311 | 1.001031 | 1.007602 | 0.009953 |
| GHR | 1.051015 | 1.002923 | 1.101414 | 0.037335 |

**Supplementary Table 5** Multivariate Cox regression analysis of ceRNAs

| id | coef | HR | HR.95L | HR.95H | P value |
| --- | --- | --- | --- | --- | --- |
| Stage | 0.505997 | 1.658638 | 1.313251 | 2.094862 | 2.16E-05 |
| age | 0.032926 | 1.033474 | 1.013998 | 1.053323 | 0.000694 |
| TPTEP1 | 0.040575 | 1.041409 | 1.011512 | 1.07219 | 0.006331 |
| LINC00461 | 1.563901 | 4.777422 | 1.569206 | 14.54479 | 0.005902 |
| PVT1 | -0.05098 | 0.950302 | 0.914694 | 0.987297 | 0.008894 |
| VCAN | 0.006247 | 1.006266 | 1.001616 | 1.010939 | 0.008219 |
| RCAN2 | 0.003675 | 1.003682 | 1.000012 | 1.007365 | 0.04923 |

**Supplementary Table 6** Multivariate Cox regression analysis of infiltrating immune cells

| id | coef | HR | HR.95L | HR.95H | P value |
| --- | --- | --- | --- | --- | --- |
| T cells CD4 memory resting | 3.599638 | 36.58499 | 1.643031 | 814.6296 | 0.022991 |
| T cells regulatory (Tregs) | 7.2543 | 1414.173 | 0.549255 | 3641082 | 0.07023 |
| T cells gamma delta | 46.92537 | 2.40E+20 | 48.4522 | 1.18E+39 | 0.032626 |
| Monocytes | 20.86355 | 1.15E+09 | 3.433335 | 3.86E+17 | 0.03724 |
| Macrophages M2 | 3.311145 | 27.4165 | 0.512842 | 1465.684 | 0.102886 |
| Dendritic cells activated | -17.3179 | 3.01E-08 | 4.62E-15 | 0.196228 | 0.030511 |
| Mast cells resting | -10.8162 | 2.01E-05 | 7.25E-09 | 0.055605 | 0.007486 |

**Supplementary Table 7** Correlation between RNAs and chemosensitivity

| Gene | Drug | cor | P value |
| --- | --- | --- | --- |
| LINC00461 | Allopurinol | -0.38267 | 0.002548 |
| LINC00461 | Mitomycin | 0.36754 | 0.003866 |
| LINC00461 | Bosutinib | -0.36696 | 0.003927 |
| LINC00461 | Dasatinib | -0.35835 | 0.004933 |
| LINC00461 | Elliptinium Acetate | 0.34344 | 0.007219 |
| LINC00461 | Pazopanib | -0.29286 | 0.023157 |
| LINC00461 | 3-Bromopyruvate (acid) | -0.29197 | 0.023598 |
| LINC00461 | Teniposide | 0.289929 | 0.024637 |
| LINC00461 | 6-Mercaptopurine | -0.28722 | 0.026073 |
| LINC00461 | Doxorubicin | 0.281361 | 0.029423 |
| LINC00461 | Trametinib | -0.2794 | 0.030622 |
| LINC00461 | Daunorubicin | 0.276195 | 0.032668 |
| LINC00461 | Cisplatin | 0.272365 | 0.035262 |
| LINC00461 | Triciribine phosphate | -0.26894 | 0.037729 |
| LINC00461 | Tyrothricin | 0.267005 | 0.039179 |
| LINC00461 | Epirubicin | 0.264692 | 0.040976 |
| LINC00461 | BN-2629 | 0.261804 | 0.043315 |
| LINC00461 | Topotecan | 0.259907 | 0.044911 |
| LINC00461 | Mitoxantrone | 0.258648 | 0.045996 |
| LINC00461 | bisacodyl, active ingredient of viraplex | -0.25523 | 0.049053 |
| LINC00461 | Irinotecan | 0.254963 | 0.049297 |
| LINC00461 | Valrubicin | 0.254933 | 0.049324 |
| PVT1 | PX-316 | -0.34279 | 0.007337 |
| PVT1 | Irofulven | 0.293565 | 0.022815 |
| PVT1 | Gemcitabine | 0.289623 | 0.024796 |
| PVT1 | Tamoxifen | -0.27023 | 0.036781 |
| PVT1 | Perifosine | -0.26967 | 0.03719 |
| PVT1 | Cladribine | 0.266114 | 0.039863 |
| PVT1 | Floxuridine | 0.262405 | 0.042819 |
| PVT1 | Ethinyl estradiol | -0.25553 | 0.048775 |
| PVT1 | Clofarabine | 0.254947 | 0.049311 |
| PVT1 | Fluphenazine | -0.25464 | 0.049596 |
| RCAN2 | Amonafide | -0.27242 | 0.035225 |
| RCAN2 | Hypothemycin | -0.26471 | 0.040958 |
| RCAN2 | PD-98059 | -0.2548 | 0.049447 |
| TPTEP1 | Axitinib | 0.530364 | 1.31E-05 |
| TPTEP1 | Nelarabine | 0.408834 | 0.001182 |
| TPTEP1 | Dexamethasone Decadron | 0.398383 | 0.001618 |
| TPTEP1 | Ponatinib | 0.38722 | 0.002239 |
| TPTEP1 | Fostamatinib | 0.374364 | 0.003211 |
| TPTEP1 | Elliptinium Acetate | 0.33224 | 0.0095 |
| TPTEP1 | Pyrazoloacridine | 0.33218 | 0.009514 |
| TPTEP1 | Bosutinib | 0.325839 | 0.011066 |
| TPTEP1 | Imatinib | 0.321264 | 0.012318 |
| TPTEP1 | Crizotinib | 0.313603 | 0.014689 |
| TPTEP1 | Irofulven | -0.29669 | 0.021343 |
| TPTEP1 | Vemurafenib | -0.28213 | 0.028962 |
| TPTEP1 | Asparaginase | 0.275476 | 0.033143 |
| TPTEP1 | Selumetinib | -0.2607 | 0.044236 |
| VCAN | Everolimus | 0.519729 | 2.09E-05 |
| VCAN | Staurosporine | 0.515432 | 2.51E-05 |
| VCAN | Ibrutinib | 0.496825 | 5.40E-05 |
| VCAN | Erlotinib | 0.473363 | 0.000134 |
| VCAN | Vandetanib | 0.456271 | 0.000248 |
| VCAN | Idelalisib | 0.45296 | 0.000279 |
| VCAN | Afatinib | 0.43143 | 0.000578 |
| VCAN | Dasatinib | 0.427332 | 0.000661 |
| VCAN | By-Product of CUDC-305 | -0.42466 | 0.00072 |
| VCAN | O-6-Benzylguanine | 0.424387 | 0.000726 |
| VCAN | Bleomycin | 0.424298 | 0.000729 |
| VCAN | Midostaurin | 0.419345 | 0.000853 |
| VCAN | Rapamycin | 0.410117 | 0.001136 |
| VCAN | Simvastatin | 0.390889 | 0.002015 |
| VCAN | Zoledronate | 0.385732 | 0.002336 |
| VCAN | Sonidegib | 0.382945 | 0.002528 |
| VCAN | Abiraterone | 0.369046 | 0.003712 |
| VCAN | Wortmannin | 0.346591 | 0.006671 |
| VCAN | AZD-9291 | 0.325158 | 0.011246 |
| VCAN | Tyrothricin | -0.32194 | 0.012127 |
| VCAN | Lapatinib | 0.316366 | 0.013793 |
| VCAN | Lapachone | -0.31352 | 0.014717 |
| VCAN | LDK-378 | -0.31138 | 0.015445 |
| VCAN | Entinostat | -0.30985 | 0.015987 |
| VCAN | Cordycepin | -0.30354 | 0.01839 |
| VCAN | Oxaliplatin | -0.29516 | 0.02205 |
| VCAN | Gefitinib | 0.293083 | 0.023049 |
| VCAN | Pentostatin | 0.291409 | 0.023881 |
| VCAN | (+)-JQ1 | 0.286207 | 0.026629 |
| VCAN | Bafetinib | -0.28176 | 0.029182 |
| VCAN | Olaparib | 0.280098 | 0.03019 |
| VCAN | Amonafide | -0.27208 | 0.035461 |
| VCAN | Tamoxifen | -0.27058 | 0.036526 |
| VCAN | Temsirolimus | 0.270313 | 0.036721 |
| VCAN | Itraconazole | 0.2671 | 0.039106 |
| VCAN | Cyclophosphamide | -0.26509 | 0.040659 |
| VCAN | Vinblastine | -0.26326 | 0.042125 |
| VCAN | Methotrexate | -0.25783 | 0.046712 |

**Supplementary Table 8** GSEA analysis of gastric cancer patients with high expression of LncRNA PVT1

| NAME | SIZE | NOM p-val | FDR q-val |
| --- | --- | --- | --- |
| KEGG_PYRIMIDINE_METABOLISM | 97 | 0 | 0.047064 |
| KEGG_SPLICEOSOME | 126 | 0 | 0.023799 |
| KEGG_CELL_CYCLE | 124 | 0 | 0.034802 |
| KEGG_NUCLEOTIDE_EXCISION_REPAIR | 44 | 0 | 0.027042 |
| KEGG_RNA_POLYMERASE | 28 | 0 | 0.02261 |
| KEGG_RNA_DEGRADATION | 57 | 0 | 0.027079 |
| KEGG_MISMATCH_REPAIR | 23 | 0.001938 | 0.022287 |
| KEGG_HOMOLOGOUS_RECOMBINATION | 28 | 0.003861 | 0.029525 |
| KEGG_PURINE_METABOLISM | 158 | 0.004132 | 0.10824 |
| KEGG_ONE_CARBON_POOL_BY_FOLATE | 17 | 0.005848 | 0.048598 |
| KEGG_DNA_REPLICATION | 36 | 0.005871 | 0.026179 |
| KEGG_BASE_EXCISION_REPAIR | 33 | 0.008016 | 0.048253 |
| KEGG_AMINOACYL_TRNA_BIOSYNTHESIS | 41 | 0.010309 | 0.052528 |
| KEGG_BASAL_TRANSCRIPTION_FACTORS | 35 | 0.015444 | 0.098876 |
| KEGG_PROTEASOME | 44 | 0.019724 | 0.088389 |
| KEGG_P53_SIGNALING_PATHWAY | 68 | 0.020121 | 0.092837 |
| KEGG_SELENOAMINO_ACID_METABOLISM | 26 | 0.034623 | 0.187902 |
| KEGG_GLYOXYLATE_AND_DICARBOXYLATE_METABOLISM | 16 | 0.036638 | 0.178257 |
| KEGG_UBIQUITIN_MEDIATED_PROTEOLYSIS | 133 | 0.038835 | 0.183115 |

Only the first 20 items are selected in the table.

**Supplementary Table 9** GSEA analysis of gastric cancer patients with low expression of LncRNA PVT1

| NAME | SIZE | NOM p-val | FDR q-val |
| --- | --- | --- | --- |
| KEGG_CALCIUM_SIGNALING_PATHWAY | 177 | 0 | 0.146709 |
| KEGG_COMPLEMENT_AND_COAGULATION_CASCADES | 69 | 0.001727 | 0.113958 |
| KEGG_HYPERTROPHIC_CARDIOMYOPATHY_HCM | 83 | 0.001927 | 0.111461 |
| KEGG_DILATED_CARDIOMYOPATHY | 90 | 0.001934 | 0.107372 |
| KEGG_ARRHYTHMOGENIC_RIGHT_VENTRICULAR_CARDIOMYOPATHY_ARVC | 74 | 0.003883 | 0.150291 |
| KEGG_NEUROACTIVE_LIGAND_RECEPTOR_INTERACTION | 271 | 0.005545 | 0.192976 |
| KEGG_O_GLYCAN_BIOSYNTHESIS | 30 | 0.015152 | 0.165874 |
| KEGG_VASCULAR_SMOOTH_MUSCLE_CONTRACTION | 114 | 0.016162 | 0.14986 |
| KEGG_ECM_RECEPTOR_INTERACTION | 84 | 0.016981 | 0.177293 |
| KEGG_ARACHIDONIC_ACID_METABOLISM | 57 | 0.018966 | 0.219908 |
| KEGG_DRUG_METABOLISM_CYTOCHROME_P450 | 70 | 0.019504 | 0.145254 |
| KEGG_PRIMARY_BILE_ACID_BIOSYNTHESIS | 16 | 0.02277 | 0.247982 |
| KEGG_GLYCOSAMINOGLYCAN_DEGRADATION | 21 | 0.025845 | 0.222592 |
| KEGG_LONG_TERM_DEPRESSION | 70 | 0.027344 | 0.280053 |
| KEGG_GLYCOSPHINGOLIPID_BIOSYNTHESIS_GANGLIO_SERIES | 15 | 0.032193 | 0.238807 |
| KEGG_ALDOSTERONE_REGULATED_SODIUM_REABSORPTION | 42 | 0.03663 | 0.226184 |
| KEGG_CELL_ADHESION_MOLECULES_CAMS | 131 | 0.046243 | 0.234113 |

Only the first 20 items are selected in the table.

**Supplementary Table 10** Go and KEGG enrichment analysis of LncRNA PVT1 related genes

| ID | Description | Count | GeneRatio | P-value | Q-value |
| --- | --- | --- | --- | --- | --- |
| GO |  |  |  |  |  |
| GO:0140097 | catalytic activity, acting on DNA | 80 | 80/1247 | 7.02E-38 | 3.79E-35 |
| GO:0140098 | catalytic activity, acting on RNA | 102 | 102/1247 | 9.23E-33 | 2.49E-30 |
| GO:0004386 | helicase activity | 63 | 63/1247 | 6.88E-31 | 1.24E-28 |
| GO:0003678 | DNA helicase activity | 42 | 42/1247 | 2.95E-27 | 3.98E-25 |
| GO:0008094 | DNA-dependent ATPase activity | 35 | 35/1247 | 1.64E-22 | 1.77E-20 |
| GO:0003697 | single-stranded DNA binding | 42 | 42/1247 | 2.99E-20 | 2.69E-18 |
| GO:0017116 | single-stranded DNA-dependent ATP-dependent DNA helicase activity | 17 | 17/1247 | 2.19E-17 | 1.48E-15 |
| GO:0043142 | single-stranded DNA-dependent ATPase activity | 17 | 17/1247 | 2.19E-17 | 1.48E-15 |
| GO:0004003 | ATP-dependent DNA helicase activity | 17 | 17/1247 | 1.07E-16 | 4.97E-15 |
| GO:0008026 | ATP-dependent helicase activity | 17 | 17/1247 | 1.07E-16 | 4.97E-15 |
| GO:0070035 | purine NTP-dependent helicase activity | 17 | 17/1247 | 1.07E-16 | 4.97E-15 |
| GO:0016887 | ATPase activity | 76 | 76/1247 | 1.10E-16 | 4.97E-15 |
| GO:0000217 | DNA secondary structure binding | 17 | 17/1247 | 5.06E-15 | 1.95E-13 |
| GO:0003688 | DNA replication origin binding | 17 | 17/1247 | 5.06E-15 | 1.95E-13 |
| GO:0140101 | catalytic activity, acting on a tRNA | 37 | 37/1247 | 1.03E-14 | 3.72E-13 |
| GO:0042393 | histone binding | 46 | 46/1247 | 2.92E-13 | 9.86E-12 |
| GO:0000400 | four-way junction DNA binding | 13 | 13/1247 | 4.56E-13 | 1.45E-11 |
| GO:0003684 | damaged DNA binding | 25 | 25/1247 | 5.27E-13 | 1.58E-11 |
| GO:0004518 | nuclease activity | 45 | 45/1247 | 5.26E-12 | 1.49E-10 |
| GO:0016796 | exonuclease activity, active with either ribo- or deoxyribonucleic acids and producing 5'-phosphomonoesters | 22 | 22/1247 | 1.21E-11 | 3.26E-10 |
| KEGG |  |  |  |  |  |
| hsa04110 | Cell cycle | 51 | 51/483 | 1.66E-28 | 2.06E-26 |
| hsa03013 | RNA transport | 59 | 59/483 | 8.90E-26 | 5.52E-24 |
| hsa03040 | Spliceosome | 50 | 50/483 | 4.89E-23 | 2.02E-21 |
| hsa03008 | Ribosome biogenesis in eukaryotes | 39 | 39/483 | 2.94E-19 | 9.14E-18 |
| hsa03030 | DNA replication | 22 | 22/483 | 1.21E-17 | 3.01E-16 |
| hsa03440 | Homologous recombination | 18 | 18/483 | 2.22E-11 | 4.59E-10 |
| hsa03460 | Fanconi anemia pathway | 18 | 18/483 | 4.72E-09 | 8.37E-08 |
| hsa03015 | mRNA surveillance pathway | 24 | 24/483 | 1.37E-08 | 2.13E-07 |
| hsa03430 | Mismatch repair | 11 | 11/483 | 6.40E-08 | 8.84E-07 |
| hsa03410 | Base excision repair | 12 | 12/483 | 6.25E-07 | 7.77E-06 |
| hsa03020 | RNA polymerase | 11 | 11/483 | 2.46E-06 | 2.78E-05 |
| hsa03018 | RNA degradation | 18 | 18/483 | 2.82E-06 | 2.92E-05 |
| hsa04115 | p53 signaling pathway | 16 | 16/483 | 1.70E-05 | 0.000163 |
| hsa04914 | Progesterone-mediated oocyte maturation | 18 | 18/483 | 8.51E-05 | 0.000755 |
| hsa03420 | Nucleotide excision repair | 11 | 11/483 | 0.000192 | 0.001528 |
| hsa00670 | One carbon pool by folate | 7 | 7/483 | 0.000197 | 0.001528 |
| hsa00240 | Pyrimidine metabolism | 12 | 12/483 | 0.000242 | 0.001765 |
| hsa04114 | Oocyte meiosis | 19 | 19/483 | 0.000798 | 0.005508 |
| hsa05014 | Amyotrophic lateral sclerosis | 40 | 40/483 | 0.000986 | 0.006449 |

Only the top 20 items are selected for KEGG and go enrichment analysis in the table.
