# Supplementary figures and images for: A novel signature based on CeRNA and immune status predicts prognostic risk and drug sensitivity in gastric cancer patients
Source: Front Immunol. 2022 Nov 22;13:951135. doi: 10.3389/fimmu.2022.951135 (PMC9723231; doi:10.3389/fimmu.2022.951135)

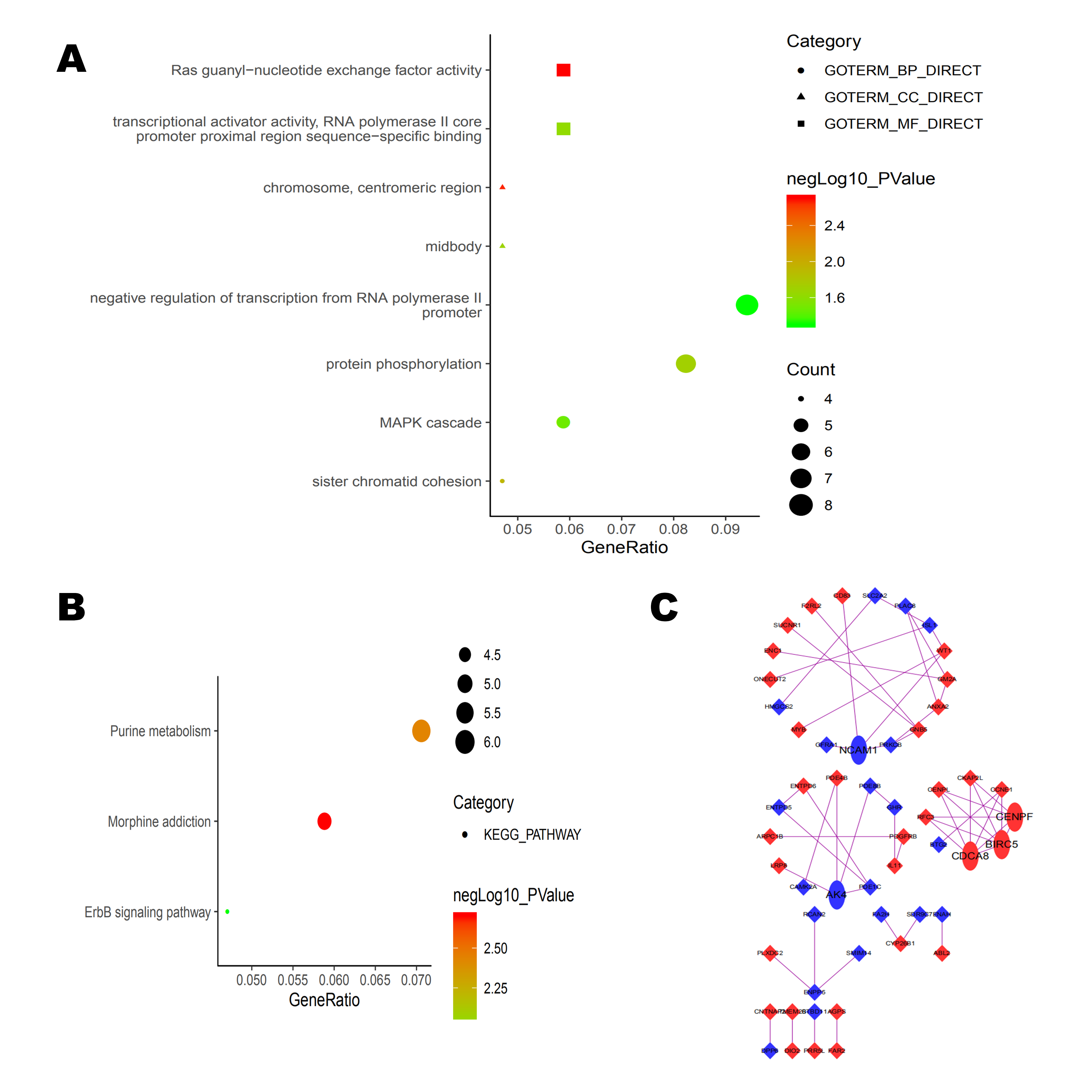

Supplement: Supplementary Figure 1 — Enrichment analysis of mRNA and construction of PPI network. (A) The GO analysis of mRNA. (B) The KEGG analysis of mRNA. (C) The protein- protein interaction network of mRNA. Red represents up regulation, and blue represents down regulation, and ellipses represent key genes. [file Image_1.tif]

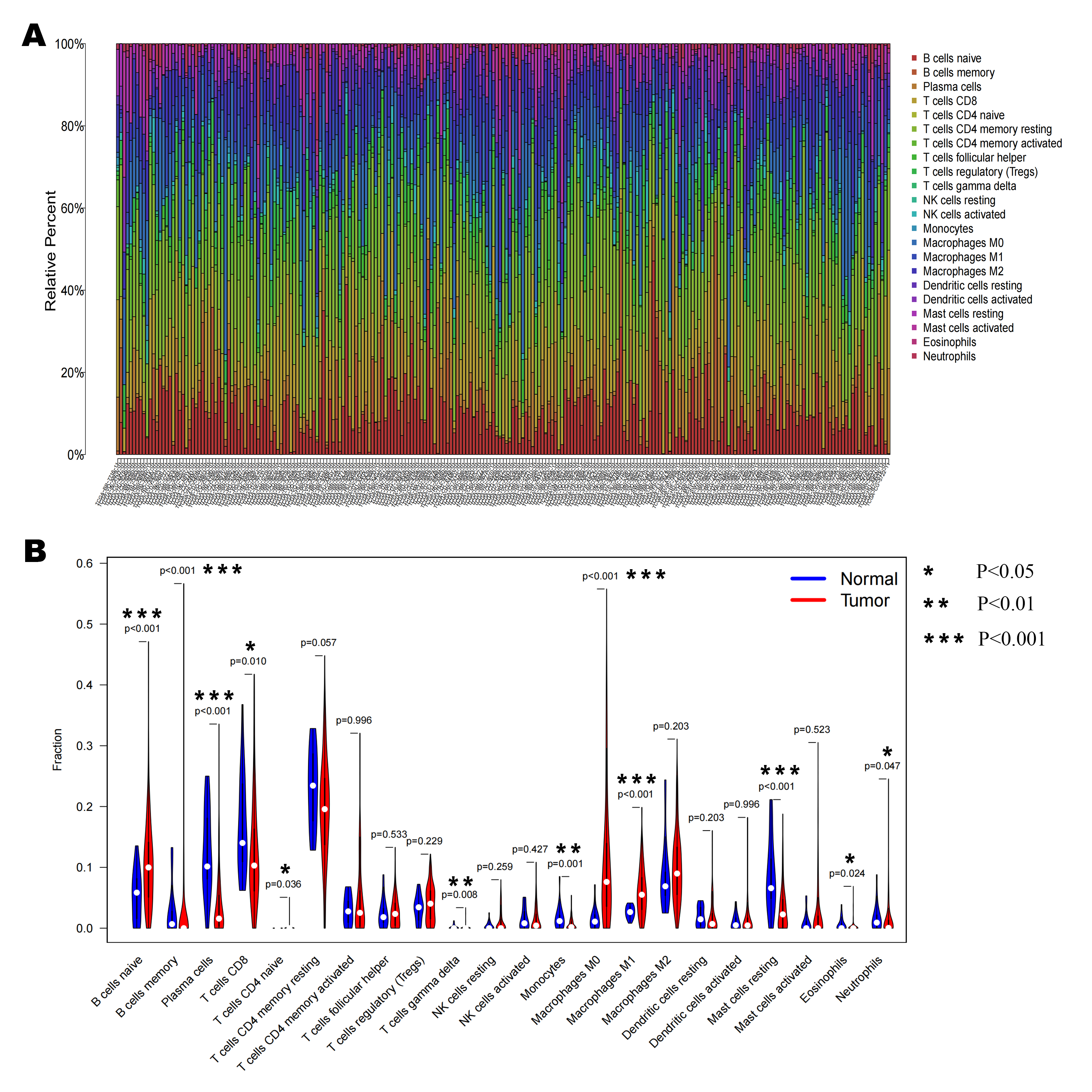

Supplement: Supplementary Figure 2 — CIBERSORT algorithm was used to predict the composition of 22 invasive immune cells in gastric cancer. (A) Abundance of 22 immune cells in gastric cancer. Violin picture (B) of the proportion of 22 immune cells in gastric cancer. [file Image_2.tif]

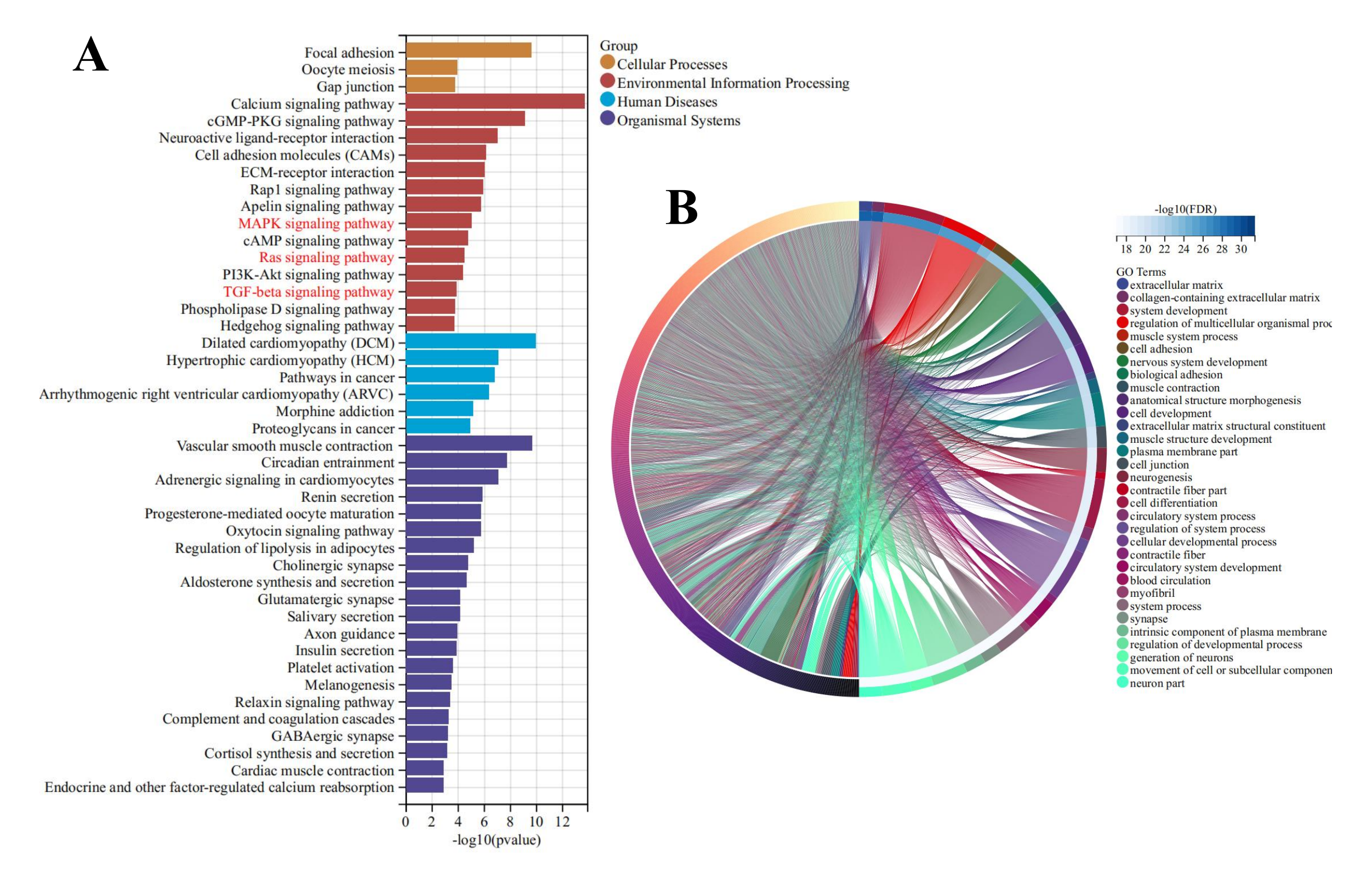

Supplement: Supplementary Figure 3 — RCAN2 (A) KEGG enrichment analysis. (B). GO enrichment analysis. [file Image_3.tif]

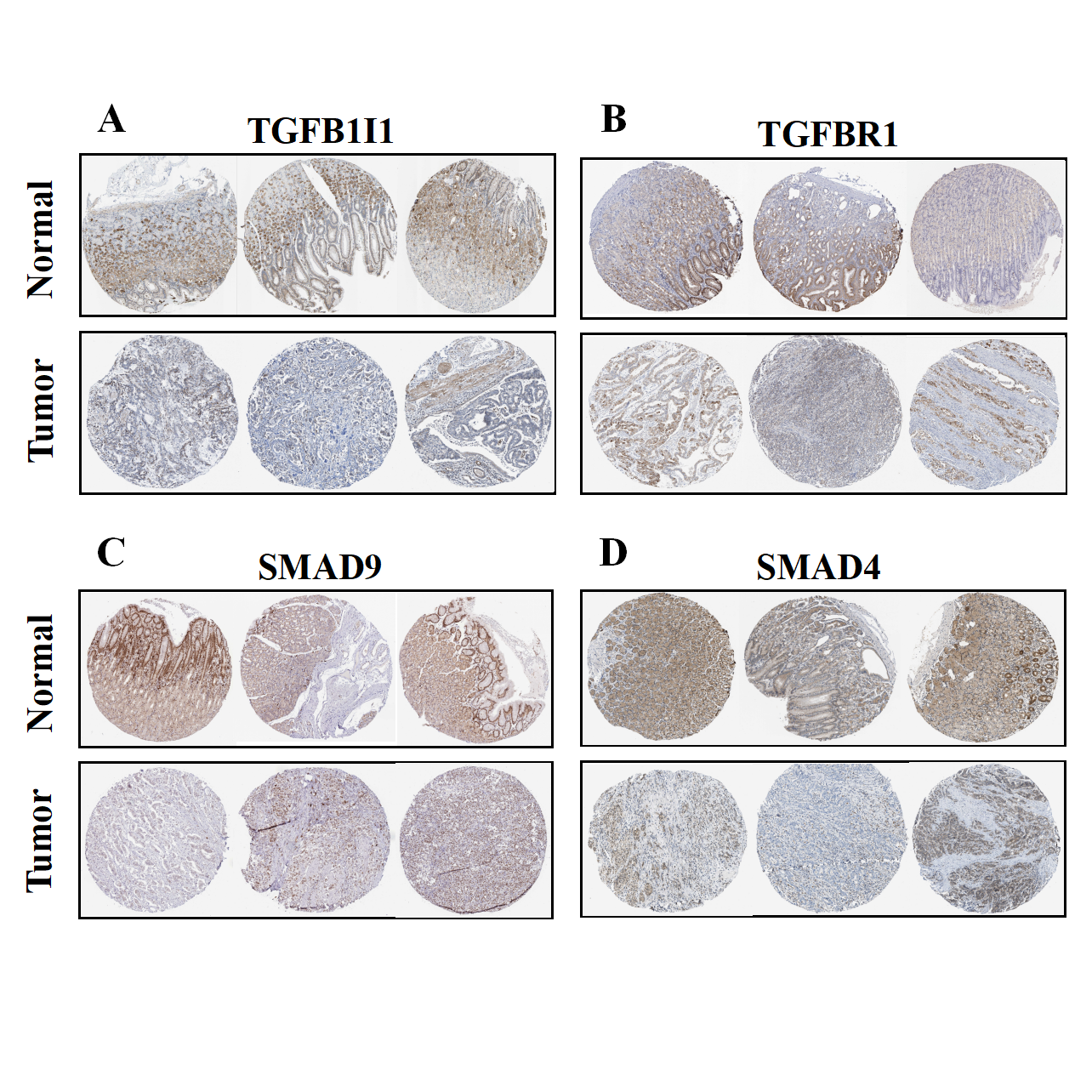

Supplement: Supplementary Figure 4 — The expression of TGF- β pathway related proteins related to RCAN2 in tumor and normal tissues. [file Image_4.tif]

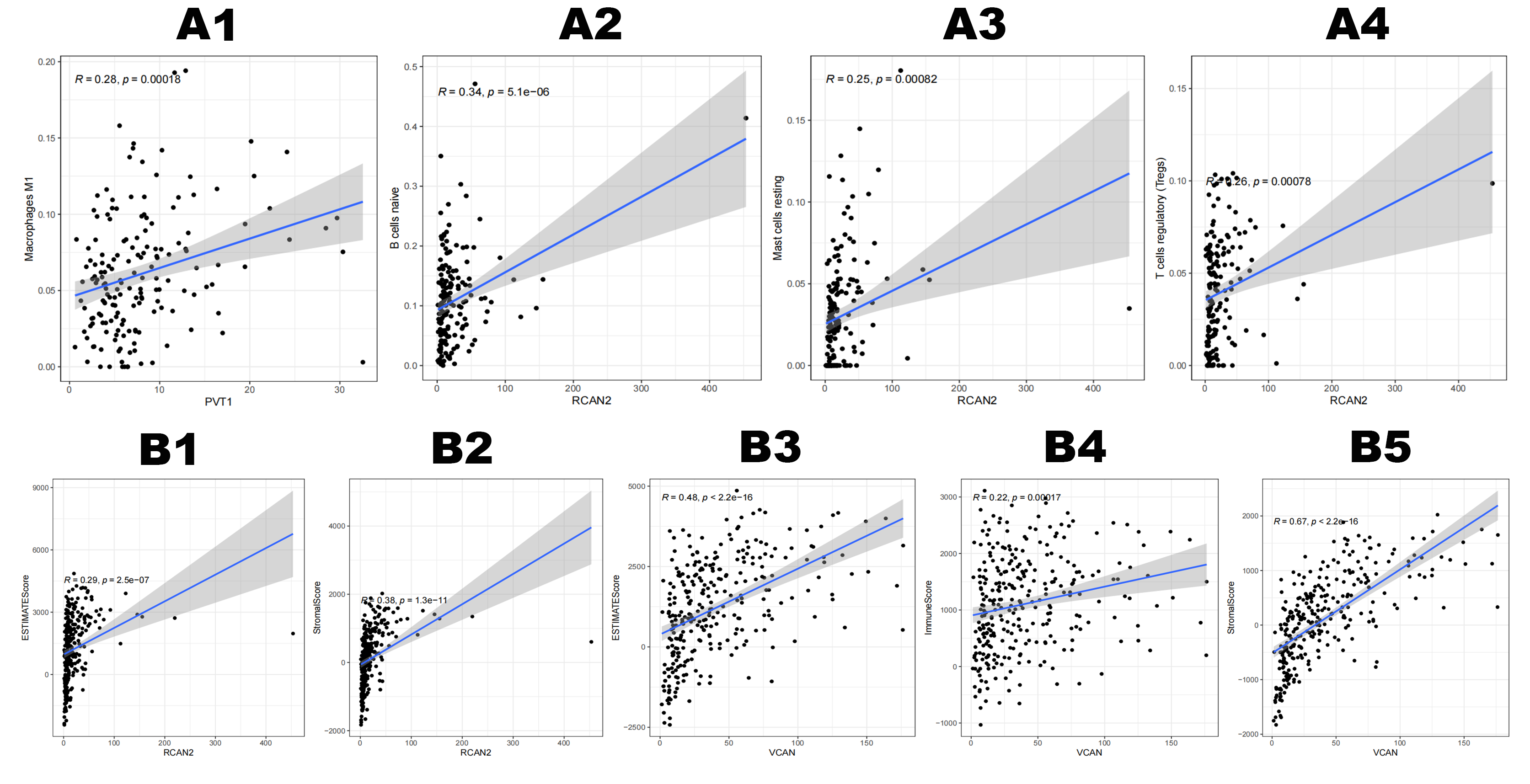

Supplement: Supplementary Figure 5 — Correlation between ceRNAs (A1-4) and immune microenvironment (B1-3) of gastric cancer. [file Image_5.tif]

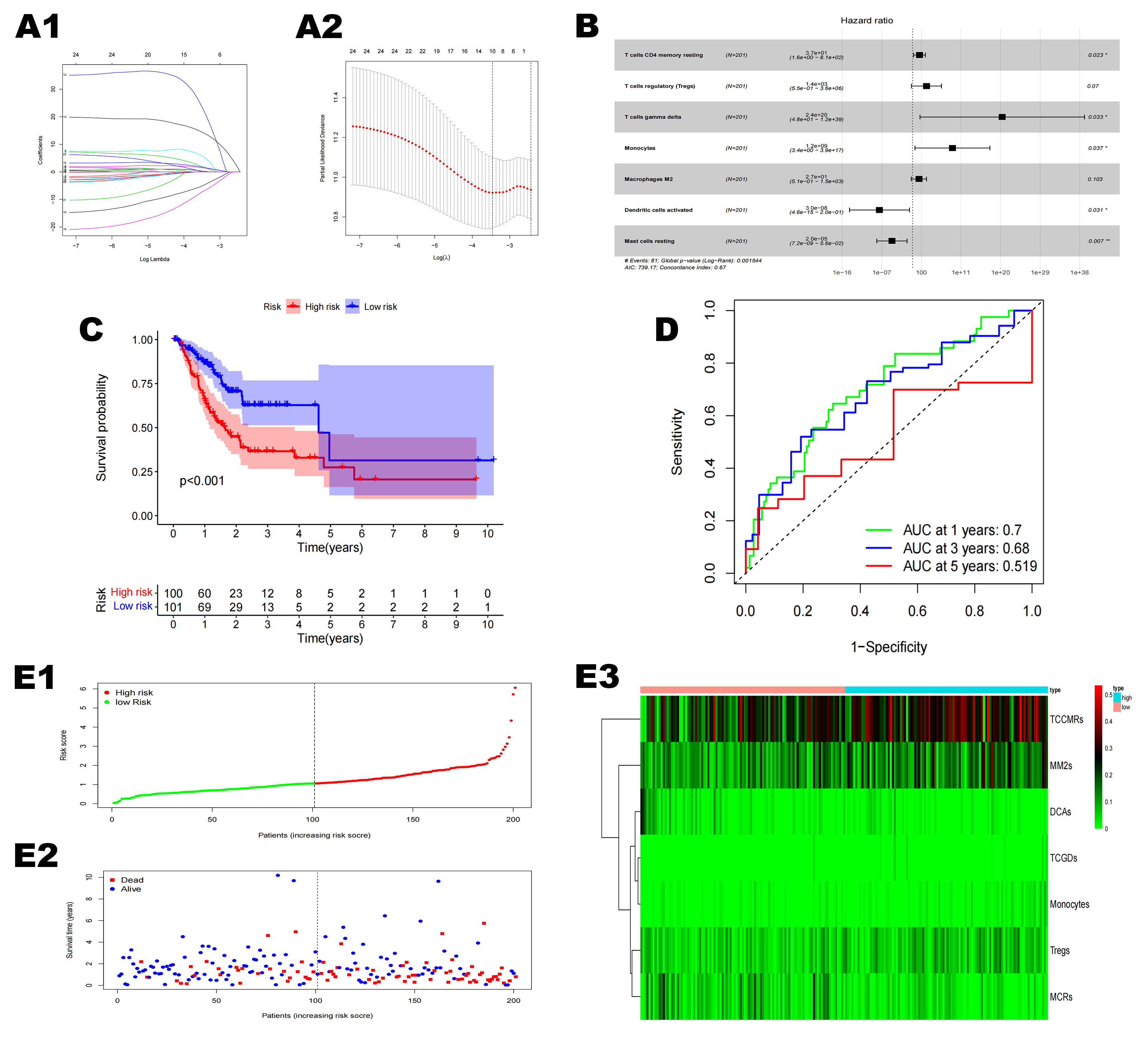

Supplement: Supplementary Figure 6 — Construction and validation of immune related diagnostic model. (A1-2) Lasso regression analysis was used to simplify the prognostic model. (B) Correlation between infiltrating immune cells and diagnosis (C) K-M survival analysis of prognostic model. (D) ROC curve of prognostic model. (E1-3) Survival status of gastric cancer patients with different risk scores. [file Image_6.tif]

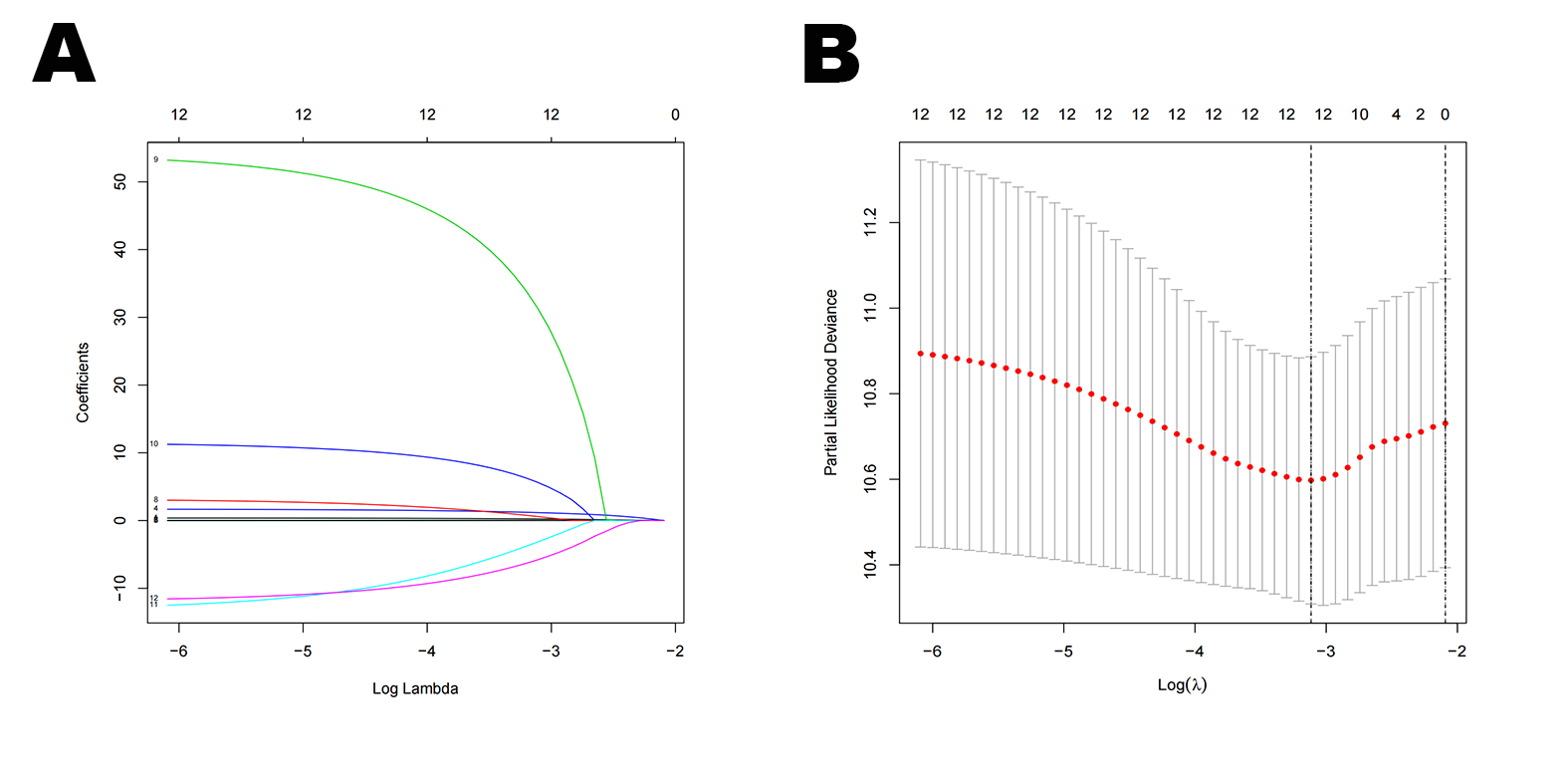

Supplement: Supplementary Figure 7 — Lasso regression analysis. (A) Use the minimum criterion of 10 times cross validation to select adjustment parameters in lasso regression. (B) Characteristic curve of lasso coefficient. [file Image_7.tif]

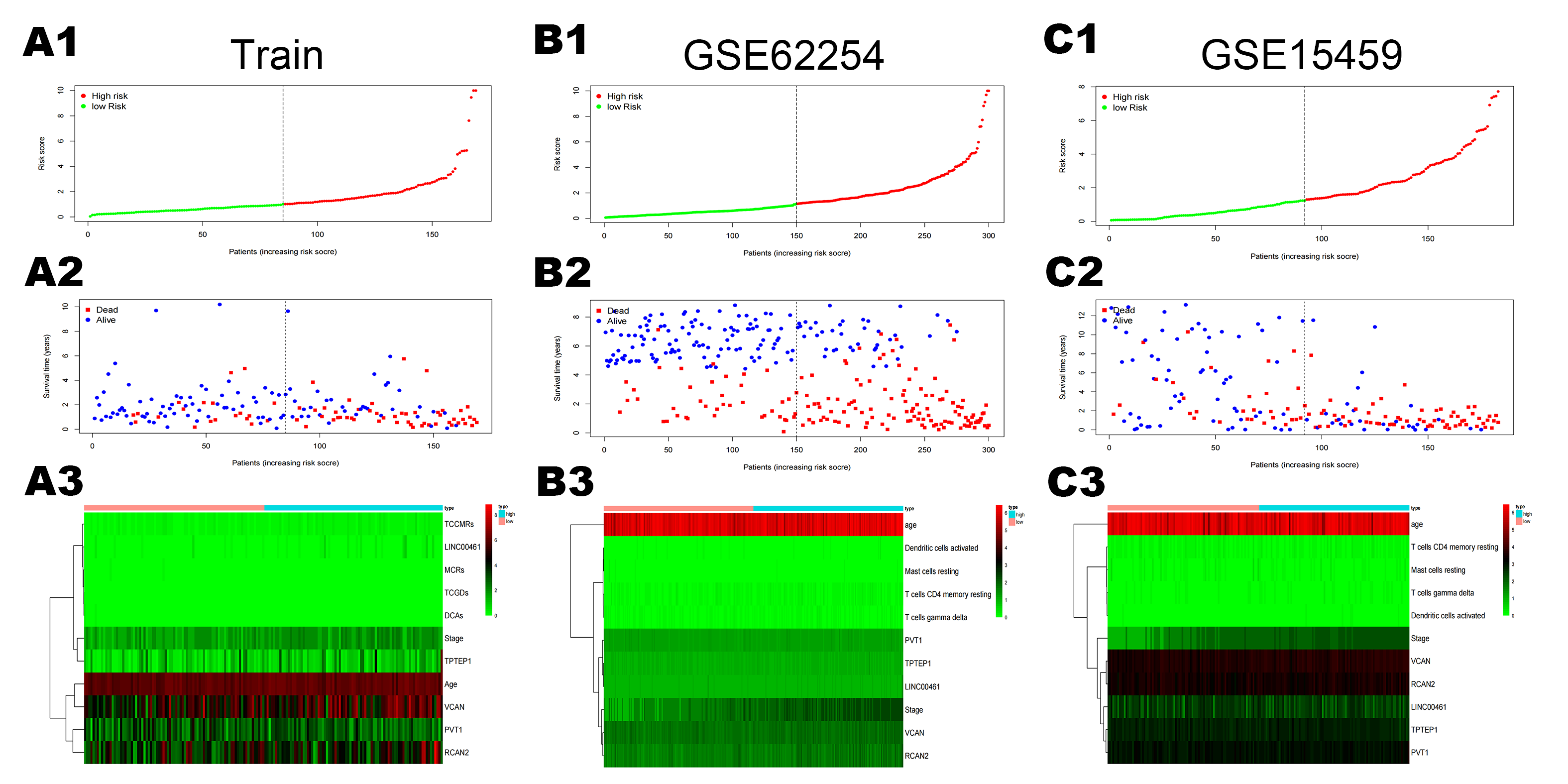

Supplement: Supplementary Figure 8 — The differences in the expression of survival status and prognostic markers of GC patients with different risk scores. (A1-3) Training set based on TCGA database. (B1-3) Verification set based on GEO62254. (C1-3) Verification set based on GEO15459. [file Image_8.tif]

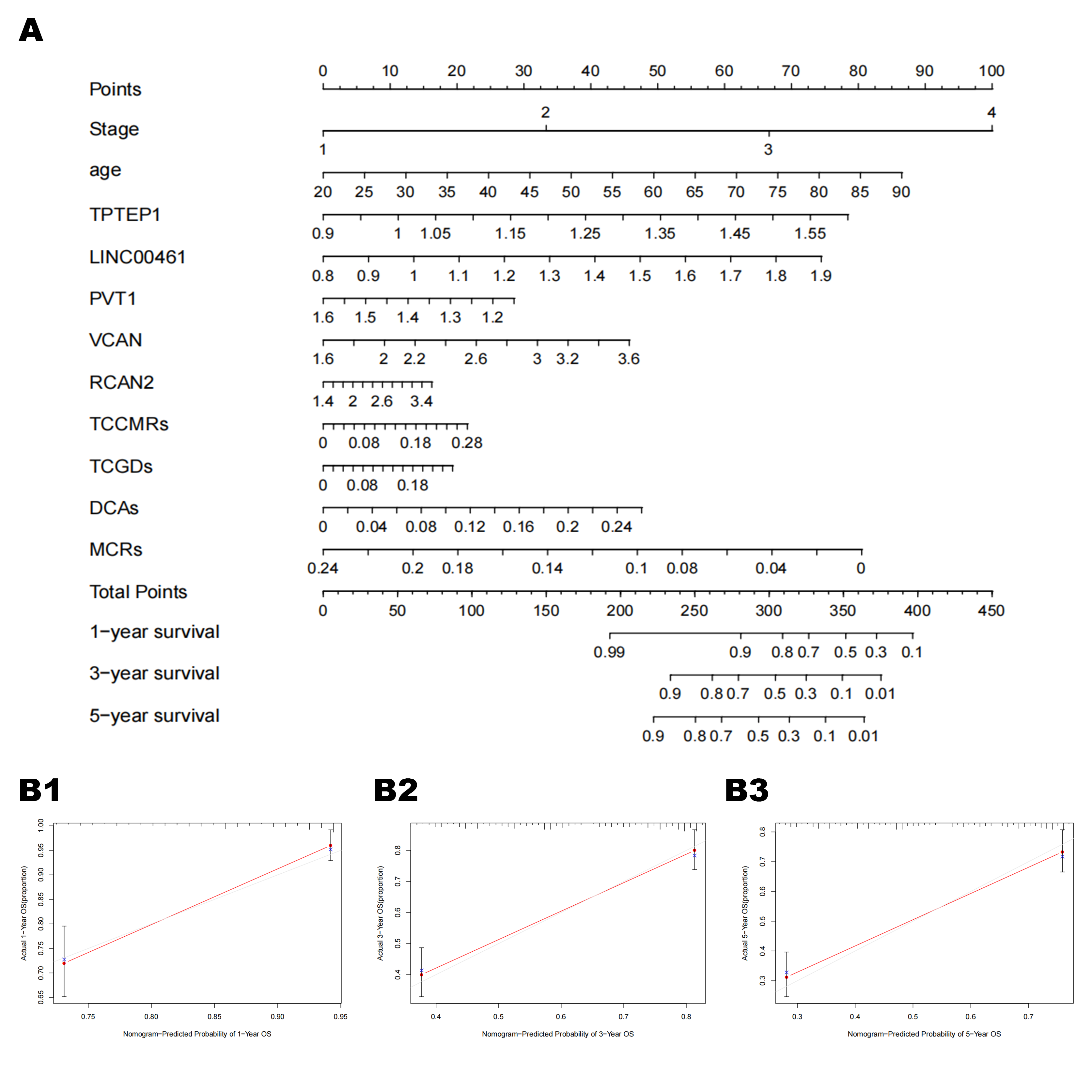

Supplement: Supplementary Figure 9 — The nomogram of the comprehensive prognostic model in the external independent validation set GSE62254. [file Image_9.tif]

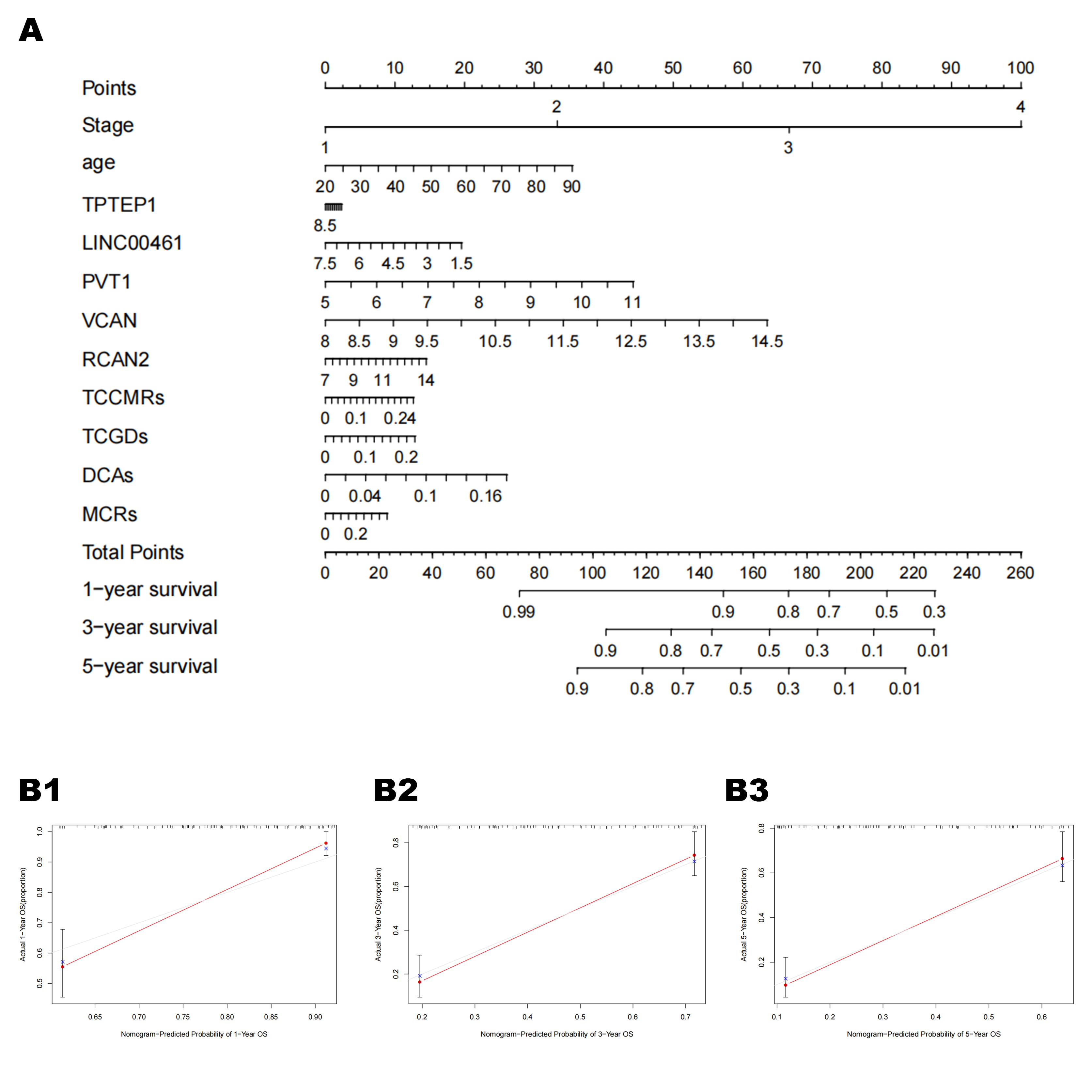

Supplement: Supplementary Figure 10 — The nomogram of the comprehensive prognostic model in the external independent validation set GSE15459. [file Image_10.tif]

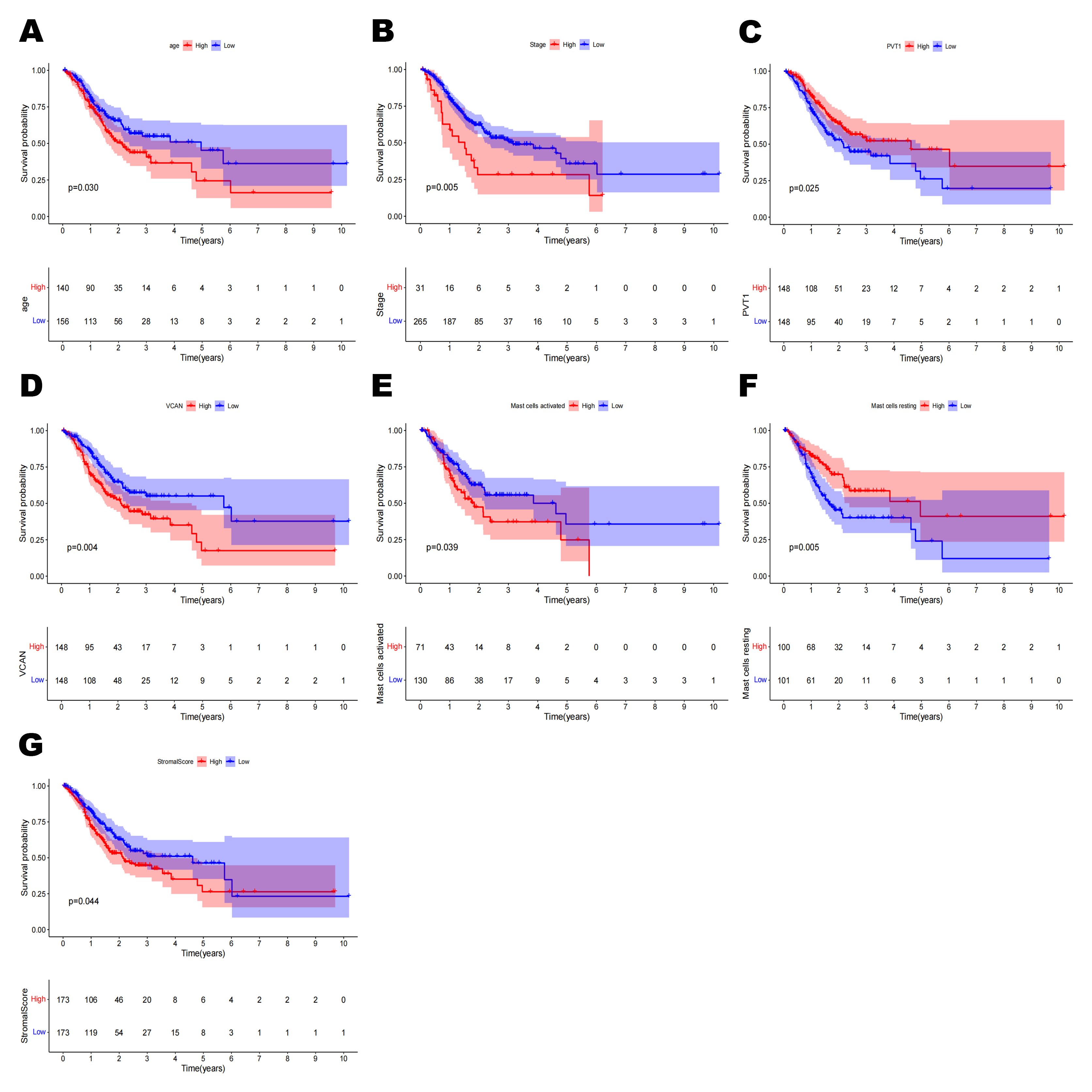

Supplement: Supplementary Figure 11 — Independent prognostic biomarkers of gastric cancer. (A) age, (B) Stage, (C) PVT1, (D) VCAN, (E) Mast cells activated, (F) Mast cells resting, (G) StromalScore. [file Image_11.tif]

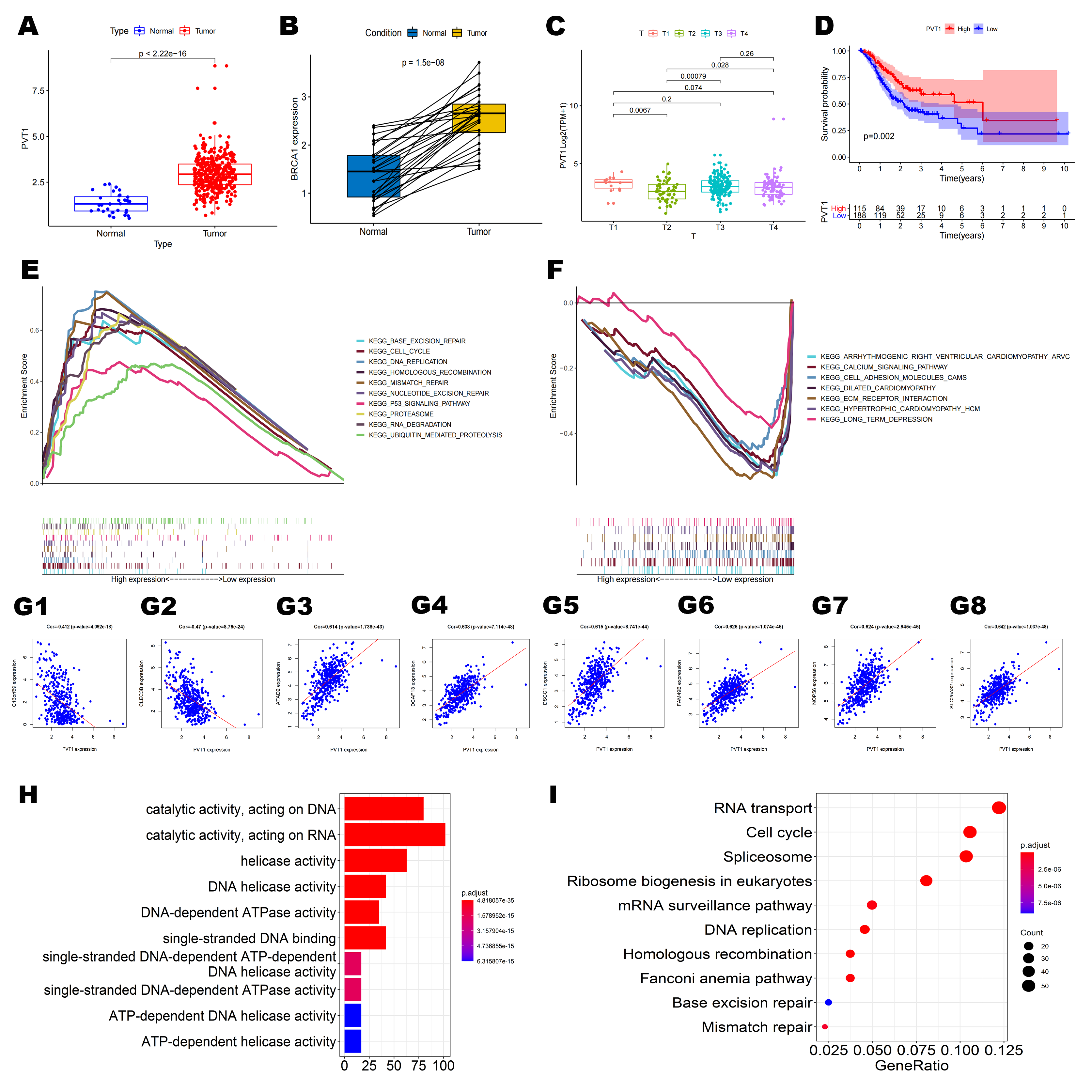

Supplement: Supplementary Figure 12 — Expression, prognosis and biological function analysis of LncRNA PVT1. (A, B) PVT1 is highly expressed in gastric cancer. (C) PVT1 is associated with T-stage of gastric cancer. (D) K-M curve showed that PVT1 was a protective factor for gastric cancer. (E) GSEA analysis of patients with high PVT1 expression. (F) GSEA analysis of patients with low PVT1 expression. (G1-8) Correlation between PVT1 and genes. (H, I) Go and KEGG enrichment analysis of PVT1. [file Image_12.tif]
